# DIACYLGLYCEROL KINASE 5 participates in flagellin-induced signaling in Arabidopsis

**DOI:** 10.1093/plphys/kiac354

**Published:** 2022-07-28

**Authors:** Tetiana Kalachova, Eliška Škrabálková, Stéphanie Pateyron, Ludivine Soubigou-Taconnat, Nabila Djafi, Sylvie Collin, Juraj Sekereš, Lenka Burketová, Martin Potocký, Přemysl Pejchar, Eric Ruelland

**Affiliations:** Institute of Experimental Botany of the Czech Academy of Sciences, Rozvojová 263, 16502 Prague, Czech Republic; Institute of Experimental Botany of the Czech Academy of Sciences, Rozvojová 263, 16502 Prague, Czech Republic; Department of Experimental Plant Biology, Charles University, Viničná 5, Prague 12844, Czech Republic; Institute of Plant Sciences Paris Saclay IPS2, CNRS, INRA, Université Paris-Sud, Université Evry, Université Paris-Saclay, Bâtiment 630, 91405 Orsay, France; Institute of Plant Sciences Paris-Saclay IPS2, Paris Diderot, Sorbonne Paris-Cité, Bâtiment 630, 91405 Orsay, France; Institute of Plant Sciences Paris Saclay IPS2, CNRS, INRA, Université Paris-Sud, Université Evry, Université Paris-Saclay, Bâtiment 630, 91405 Orsay, France; Institute of Plant Sciences Paris-Saclay IPS2, Paris Diderot, Sorbonne Paris-Cité, Bâtiment 630, 91405 Orsay, France; Laboratoire de Physiologie Cellulaire et Moléculaire des Plantes, Sorbonne Université, F-75005 Paris, France; Laboratoire de Physiologie Cellulaire et Moléculaire des Plantes, Sorbonne Université, F-75005 Paris, France; Institute of Experimental Botany of the Czech Academy of Sciences, Rozvojová 263, 16502 Prague, Czech Republic; Institute of Experimental Botany of the Czech Academy of Sciences, Rozvojová 263, 16502 Prague, Czech Republic; Institute of Experimental Botany of the Czech Academy of Sciences, Rozvojová 263, 16502 Prague, Czech Republic; Institute of Experimental Botany of the Czech Academy of Sciences, Rozvojová 263, 16502 Prague, Czech Republic; CNRS Enzyme and Cell Engineering Laboratory, Université de Technologie de Compiègne, Rue du Docteur Schweitzer, 60203 Compiègne, France

## Abstract

Flagellin perception is a keystone of pattern-triggered immunity in plants. The recognition of this protein by a plasma membrane (PM) receptor complex is the beginning of a signaling cascade that includes protein phosphorylation and the production of reactive oxygen species (ROS). In both Arabidopsis (*Arabidopsis thaliana*) seedlings and suspension cells, we found that treatment with flg22, a peptide corresponding to the most conserved domain of bacterial flagellin, caused a rapid and transient decrease in the level of phosphatidylinositol (PI) 4,5-bisphosphate along with a parallel increase in phosphatidic acid (PA). In suspension cells, inhibitors of either phosphoinositide-dependent phospholipases C (PLC) or diacylglycerol kinases (DGKs) inhibited flg22-triggered PA production and the oxidative burst. In response to flg22, receptor-like kinase-deficient *fls2*, *bak1*, and *bik1* mutants (FLAGELLIN SENSITIVE 2, BRASSINOSTEROID INSENSITIVE 1-associated kinase 1, and BOTRYTIS-INDUCED KINASE 1, respectively) produced less PA than wild-type (WT) plants, whereas this response did not differ in NADPH oxidase-deficient *rbohD* (RESPIRATORY BURST OXIDASE HOMOLOG D) plants. Among the DGK-deficient lines tested, the *dgk5.1* mutant produced less PA and less ROS after flg22 treatment compared with WT seedlings. In response to flg22, *dgk5.1* plants showed lower callose accumulation and impaired resistance to *Pseudomonas syringae* pv. *tomato* DC3000 *hrcC-*. Transcriptomics revealed that the basal expression of defense-related genes was altered in *dgk5.1* seedlings compared with the WT. A GFP-DGK5 fusion protein localized to the PM, where RBOHD and PLC2 (proteins involved in plant immunity) are also located. The role of DGK5 and its enzymatic activity in flagellin signaling and fine-tuning of early immune responses in plant–microbe interactions is discussed.

## Teaser

Arabidopsis DIACYLGLYCEROL KINASE 5 activation during plant–microbe recognition is required for an appropriate immune response.

## Introduction

In recent years, membrane phospholipids have been shown to be key players in plant responses to environmental stresses. Under stress conditions, the composition of cell membranes can fluctuate, with a stimulated cell being able to quickly adapt and transmit a stress signal to neighboring cells. This membrane plasticity involves the rapid turnover of different types of phospholipids that can be phosphorylated, dephosphorylated, or cleaved by the activation of phospholipases and lipid-kinases (Tjellström et al., 2008; Noack and Jaillais, 2020). In parallel, phospholipids trigger numerous signaling pathways during immunity (Abd-El-Haliem and Joosten, 2017; Cacas et al., 2017; Krčková et al., 2018; Gully et al., 2019), hormonal transduction (Munnik and Testerink, 2009; Janda et al., 2013; Ruelland et al., 2015; Kalachova et al., 2016), and response to abiotic stresses (Vergnolle et al., 2005; Krčková et al., 2015; Pejchar et al., 2015; Hou et al., 2016). However, a detailed mechanistic understanding of the connections between phospholipids and other molecular components is still missing.

The simplest phospholipid, phosphatidic acid (PA), is composed of diacylglycerol (DAG) with a phosphoryl group esterified on the *sn*-3 hydroxyl of the glycerol chain. PA is produced by two pathways: (i) from structural lipids cleaved by phospholipase D (PLD) and (ii) from DAG phosphorylated by DAG kinase (DGK). In the latter case, DAG may be generated either by the activity of phosphoinositide-specific phospholipase C (PI-PLC) that uses phosphoinositides (phosphorylated forms of PI) as a substrate or by non-specific phospholipases C (NPCs) that use structural phospholipids. In plants, PA levels have been shown to change under different stress conditions, including pathogen attack and abiotic stresses such as cold temperature or drought stress (Ruelland et al., 2015; Pokotylo et al., 2018).

PA accumulation occurs in response to many pathogen-associated molecular patterns (PAMPs) (xylanase, flagellin, cryptogein, and chitosan) in several independent plant models (van der Luit et al., 2000; de Jong et al., 2004; Laxalt et al., 2007; Cacas et al., 2017). PLD, PI-PLC, and DGK activities and encoding genes have been shown to be upregulated in several experiments dealing with immunity (Ruelland et al., 2015). Signaling mediators of lipid origin, such as PA or phosphoinositides, can physically interact with proteins and so far 35 PA-interacting plant proteins have been identified (Delage et al., 2012; Pleskot et al., 2012; Hou et al., 2016; Putta et al., 2016; Pokotylo et al., 2018).

Some of these PA-binding proteins are known to be involved in the first layers of plant immunity and includes NADPH-oxidase RESPIRATORY BURST OXIDASE HOMOLOGUE protein D (RBOHD) (Zhang et al., 2009; Kadota et al., 2015; Cacas et al., 2017). RBOHD is a key component of apoplastic reactive oxygen species (ROS) production involved in the fast oxidative burst after pathogen recognition (Torres et al., 2002; Jones and Dangl, 2006). At the same time, RBOHD can be activated by PA as a part of a hormonal stress signal transduction cascade induced by abscisic acid (ABA) (Zhang et al., 2009) and salicylic acid (SA) (Kalachova et al., 2013). A secondary outcome of PA production via the PI-PLC/DGK pathway is the decrease of phosphoinositide levels. Phosphoinositides are involved in maintaining the transcriptome of resting cells and modulating the transcriptome in response to phytohormones (Kalachova et al., 2015). Indeed, the majority of genes commonly regulated by ABA and SA show phosphoinositide-dependent expression (Kalachova et al., 2016). Furthermore, phosphoinositides are responsible for vesicular trafficking required for the correct translocation of plasma membrane (PM) PAMP receptors (Antignani et al., 2015).

A commonly studied PAMP is bacterial flagellin (Chinchilla et al., 2007), a conserved bacterial flagella protein that is present in the Arabidopsis (*Arabidopsis thaliana*) leaf pathogen *Pseudomonas syringae* pv. *tomato* (*Pst*) (Albert, 2013). In a simplified model system, the response to flagellin is studied using flg22 (QRLSTGSRINSAKDDAAGLQIA), a synthetic 22 amino acid peptide representing the N-terminal epitope responsible for recognition by the FLAGELLIN SENSITIVE 2 (FLS2) receptor-BRASSINOSTEROID INSENSITIVE 1-associated kinase 1 (BAK1) receptor complex and for launching PAMP-triggered immunity (PTI). According to the classical scheme, flg22 binding induces heteromerization of FLS2 with BAK1, and their reciprocal activation (Gómez-Gómez and Boller, 2000; Chinchilla et al., 2006, 2007; Heese et al., 2007; Sun et al., 2013). The receptor-like cytoplasmic kinase BOTRYTIS-INDUCED KINASE 1 (BIK1) is another component of this immune receptor complex. BIK1 is an N-myristoylated protein that is stably associated with FLS2 at the PM even in the absence of PAMP treatment (Veronese et al., 2006). It becomes activated upon co-receptor (BAK1) recruitment and subsequently partially dissociates from the complex. BIK1 then interacts specifically with the N-terminus of RBOHD, where it phosphorylates regulatory sites that are required and sufficient for RBOHD activation (Kadota et al., 2014; Li et al., 2014).

We here investigated the role of DGKs in flagellin transduction in Arabidopsis cell suspension and seedlings. Phospholipid turnover after flg22 treatment, ROS production, callose deposition, and transcriptome remodeling in suspension cell cultures and in seedlings were analyzed. In both systems, a rapid production of PA via the PI-PLC/DGK pathway, involving DGK5, was identified. A T-DNA null mutant *dgk5.1* showed impaired responses to flg22 and enhanced susceptibility to *Pst hrcC-*, thus inferring the importance of DGK5 in PTI. Transient expression in *Nicotiana benthamiana* revealed the PM localization of DGK5, PLC2, and RBOHD. Our data strongly suggest that lipid signaling involving DGK5 is a component of the early steps of flagellin perception and the establishment of PTI.

## Results

### flg22 induces a rapid and reversible PA accumulation and a PIP_2_ decrease in suspension cells

**Figure 1:**
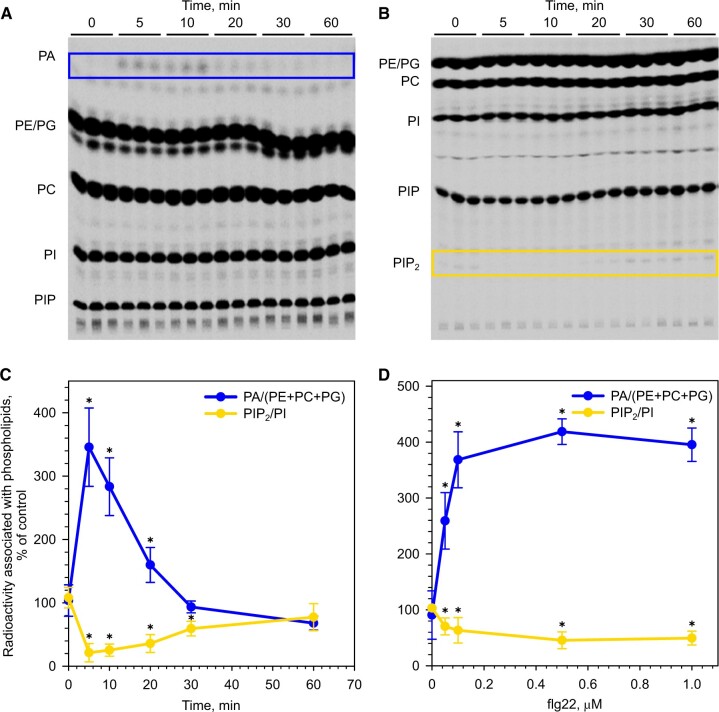
Treatment of Arabidopsis suspension cells with flg22 induces a transient increase in PA and a decrease in PIP_2_ levels. Seven-day-old suspension cells were treated with flg22. Lipids were extracted at different time points after flg22 treatment. ^33^P-orthophosphate was added 20 min before lipid extraction. A, TLC separation of ^33^P-labeled phospholipids in an acidic solvent system. B, TLC separation of phospholipids in an alkaline solvent system. C, Time-course response after the addition of 100 nM flg22, radioactivity associated with PA (blue line) and PIP_2_ (yellow line). D, Relative radioactivity associated with PA (blue line) and PIP_2_ (yellow line) after a 10-min treatment with different flg22 concentrations. Radioactivity associated with PA was normalized to that in phosphatidylethanolamine (PE), phosphatidylcholine (PC), phosphatidylglycerol (PG); radioactivity associated with PIP_2_ was normalized to that in PI. Obtained values were normalized to controls. Data are presented as means ± se. Asterisks denote samples significantly different from controls, *P* < 0.05, paired *t* test, *n* = 3–4.

The effects of flg22 on phospholipid turnover were investigated in 7-day-old cells of a suspension culture. Cells were treated with flg22 and lipids were extracted at different time points. ^33^P-orthophosphate was added 20 min before lipid extraction. Lipids were separated by thin-layer chromatography (TLC) developed in acidic (Figure 1A) and alkaline (Figure 1B) solvent systems. The level of radioactivity associated with PA was normalized to that in structural lipids and radioactivity associated with PI 4,5-bisphosphate (PIP_2_) was normalized to that in PI.

A rapid 3.5-fold increase in the relative radioactivity associated with PA was observed after a treatment with 100 nM flg22. This was paralleled with a decrease in the radioactivity associated with PIP_2_ (Figure 1C). These phospholipid changes were transient, and after 20–30 min the radioactivity levels in PA and PIP_2_ returned to control levels. A dose of 50 nM flg22 was sufficient to induce significant changes in PA and PIP_2_ levels (Figure 1D). The rapid and opposite effects of flg22 on PA and PIP_2_ labeling suggested a metabolic pathway in which PA was produced by DGKs that catalyze the phosphorylation of DAG, that is formed as a result of the action of PI-PLC.

Whether the detected PA is preferentially produced from either PI-PLC/DGK or PLD activities was distinguished using different labeling times. Indeed, radioactive ^33^P-orthophosphate added to the cultivation media is rapidly metabolized to ^33^P-ATP. So, in short labeling conditions (of 15–30 min), processes connected to ATP are mainly observed. Therefore, PA detected in treated, short-labeled cells was more likely to be produced via the PI-PLC/DGK pathway, and—more precisely—as a result of DAG phosphorylation (Ruelland et al., 2002). Longer incubations with ^33^P-orthophosphate allow the labeling of structural lipids and therefore the contribution of PLD to PA production can be evaluated. A significant difference in relative PA content between flg22-treated and control cells was observed only in short (20 min) labeling conditions (Supplemental Figure S1), thus indicating that the activity of PI-PLC/DGK was mainly responsible for the production of labeled PA. With a longer labeling time, no difference in PA production was seen between flg22-treated and control cells, which implies that the contribution of PLD to the PA accumulation in response to flg22 in the short-time labeling was negligible.

### flg22-triggered PA production involves the DGK pathway

**Figure 2:**
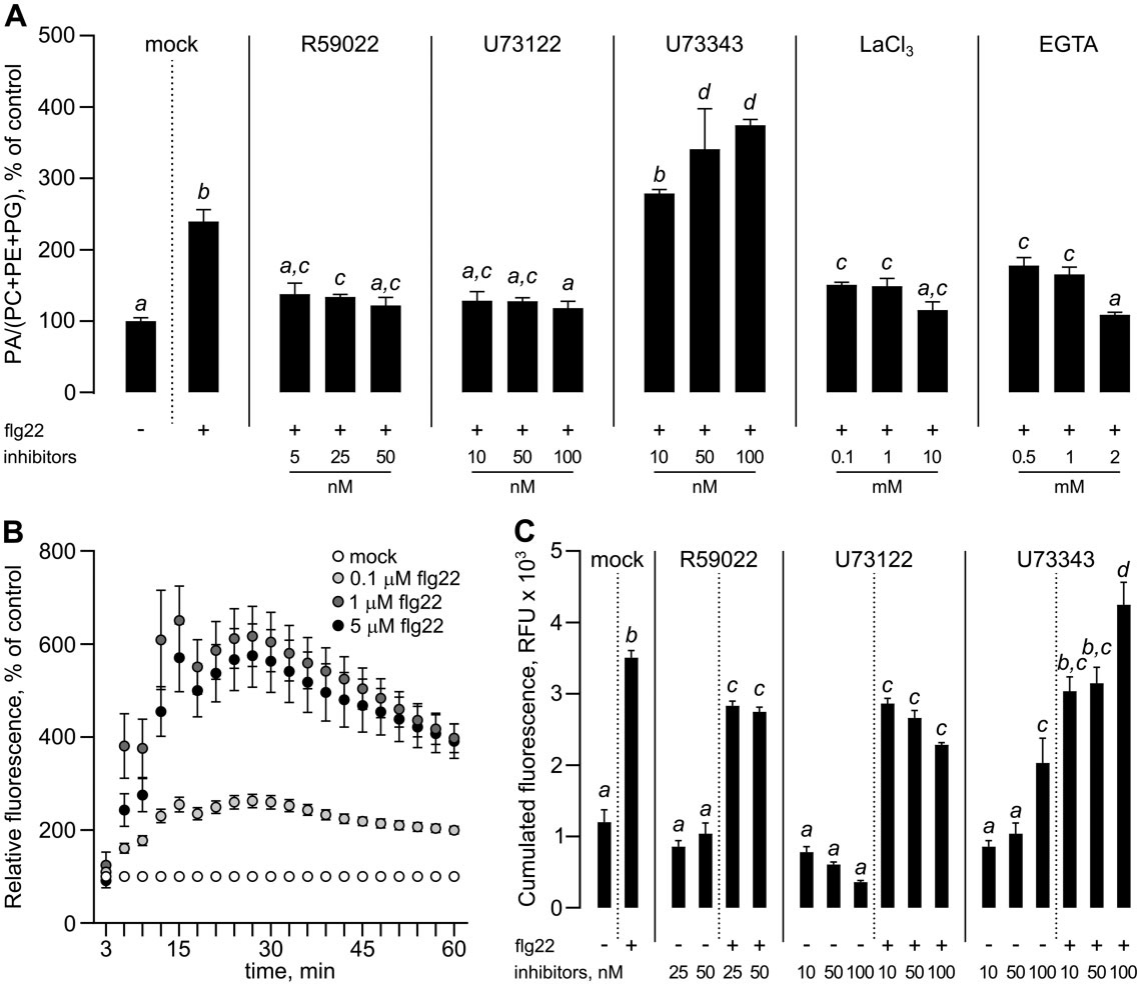
Chemical inhibition of DGK and phosphoinositide-specific PLC (PI-PLC) affects PA accumulation and ROS production after flg22 treatment in Arabidopsis suspension cells. A, Formation of PA after 10 min of 0.1 µM flg22 treatment in the presence of lipid signaling inhibitors: DGK inhibitor I (R59022); PI-PLC inhibitor U73122; or its biologically inactive analog U73343, LaCl_3_, and EGTA. Experiments were performed on suspension cells, 7 days after subculturing. ^33^P-orthophosphate was added 20 min before lipid extraction and inhibitors were added 30 min before flg22 addition. Lipids were extracted 10 min after flg22 treatment and separated by TLC using an acidic solvent system. The radioactivity associated with PA was normalized to that in structural phospholipids and the obtained values were then normalized to that in controls. Data are presented as means ± se, different letters mean significant difference (one-way ANOVA, Tukey’s HSD, *P* < 0.05, *n* = 5). PC, phosphatidylcholine; PE, phosphatidylethanolamine and PG, phosphatidylglycerol. B, Kinetics of ROS formation after flg22 treatment, dose response. Cell suspensions were labeled with 5.8 µM 2′,7′-dichlorodihydrofluorescein diacetate (H2DCFDA) in microplates for 15 min and different concentrations of flg22 were then added. Fluorescence was measured every 3 min. Inhibitors were injected 30 min prior to flg22. Data correspond to H2DCFDA fluorescence normalized to mock-treated controls. C, The effect of lipid signaling inhibitors on ROS formation. Cumulated fluorescence of H2DCFDA-labeled cells after 60 min of 1 µM flg22 treatment expressed as relative fluorescence units (RFU) is shown. Data are presented as means ± se. Different letters indicate statistically significant different values (one-way ANOVA, Tukey’s HSD, *P* < 0.05, *n* = 5).

A contribution of the PI-PLC/DGK pathway to the observed PA production was also assessed by a pharmacological approach. DGK inhibitor I (or R59022) impaired the accumulation of PA in response to flg22 (Figure 2A). The commonly used inhibitor of PI-PLC activity, U73122, was then tested. PA accumulation was impaired in the presence of 10 µM U73122, while its biologically inactive analog U73343 had no significant impact. Interestingly, the addition of LaCl_3_, an inhibitor of Ca^2+^ plasmalemma channels, or the Ca^2+^ chelator EGTA (ethylene glycol-bis(β-aminoethyl ether)-*N,N,N′,N′*-tetraacetic acid) inhibited the accumulation of labeled PA. This is in agreement with the calcium dependency of PI-PLC and points to calcium being upstream to PA accumulation in response to flg22. This unambiguously showed that PI-PLC activity coupled to DGK led to the production of PA in response to flg22 in Arabidopsis suspension cells.

### flg22-triggered PA formation participates in ROS production

To study whether inhibitors of PI-PLC and DGK activities influence flg22-triggered ROS formation, cell suspensions were labeled with 2′,7′-dichlorodihydrofluorescein diacetate (H2DCFDA) in microplates for 15 min. Different concentrations of flg22 peptide were then added and fluorescence was measured every 3 min. A strong seven-fold increase of ROS levels was detected after a 1-µM flg22 treatment (Figure 2B), and this concentration was selected to test inhibitors which were added 30 min prior to flg22. In the absence of flg22, the inhibitors did not interfere with ROS-associated fluorescence except for 100 μM U73343 where H2DCFDA fluorescence was seen to be slightly enhanced. The inhibitors, R59022 and U73122, led to a reduced amount of ROS produced 60 min after flg22 treatment, while U73343 showed no effect (Figure 2C). These observations clearly establish the involvement of the PI-PLC/DGK pathway in flg22-triggered ROS generation.

### Flg22 induces PA production in Arabidopsis seedlings downstream of the FLS2-BAK1 receptor complex, but upstream of RBOHD

**Figure 3:**
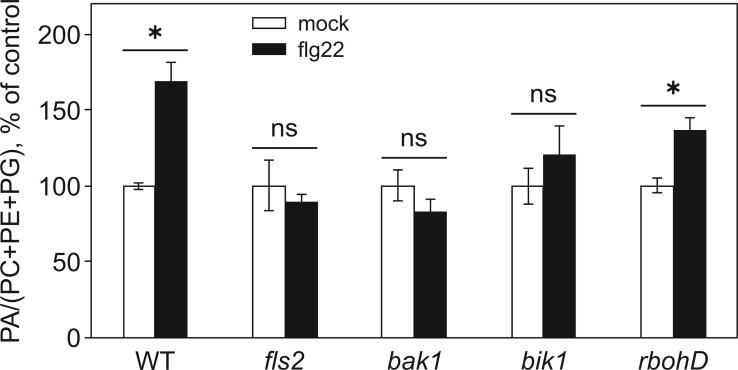
PA accumulation in WT and mutants deficient in different protein kinases and NADPH oxidase. Arabidopsis WT, *fls2*, *bak1*, *bik1*, and *rbohD* seedlings were labeled with ^33^Pi for 1 h, then treated with 500 nM flg22. Lipids were separated by TLC using an acidic solvent system. PA content was normalized to structural phospholipids and the obtained values were normalized to control values for each genotype. Statistical analysis was done by Kruskal–Wallis one-way ANOVA followed by post hoc pairwise comparisons. Data are presented as means ± se. Asterisks denote statistically significant difference between variants, *P* < 0.05, *n* = 2–11; ns, not significant.

Next, the possible interplay of lipid signaling enzymes with known components of flagellin recognition and early signaling was investigated. Arabidopsis seedlings were used because of the availability of the necessary T-DNA mutants. Eleven-day-old seedlings grown in vitro in liquid medium were used in all further experiments. The concentration dependence and dynamics of PA accumulation in response to flg22 were determined, using optimized labeling conditions for seedlings. The dynamics of PA in flg22-treated seedlings appeared to be similar to that of cell suspensions and showed a peak between 5 and 10 min; however, the decrease back to the control level was slower than in suspension cells (Supplemental Figure S2). A 10-min treatment with 500 nM flg22 was used in all further seedling experiments unless otherwise stated. The PA response to flg22 was studied in Arabidopsis mutant lines for FLS2, BAK1, BIK1, and RBOHD. Importantly, the *fls2*, *bak1*, and *bik1* mutant plants did not show a PA increase in response to flg22 (Figure 3). On the contrary, flg22-treated *rbohD* mutant plants accumulated PA in a manner comparable to WT plants (Figure 3). Together with our data on suspension cells, this provides evidence that PA production is occurring upstream of NADPH-oxidases.

### DGK5 plays a role in flg22-induced PA formation, ROS production, and callose deposition

**Figure 4:**
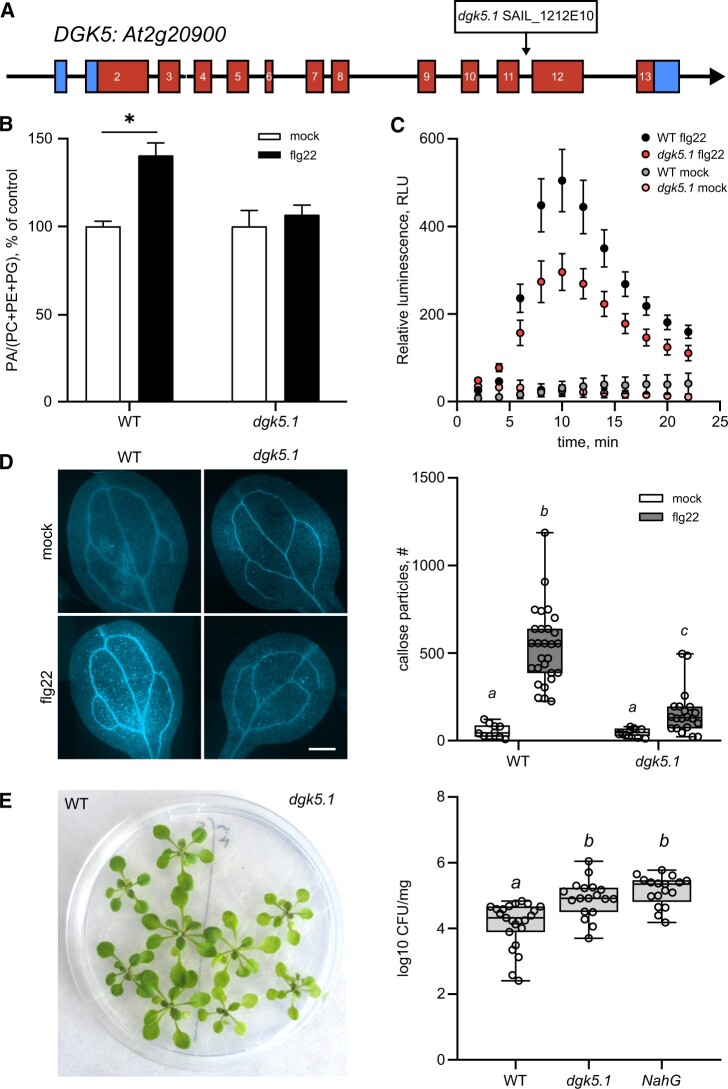
PTI-associated responses in *dgk5.1* seedlings. A, Arabidopsis DGK5 gene structure and the position of the T-DNA insertion in the *dgk5.1* mutant. B, PA accumulation after flg22 treatment. Eleven-day-old seedlings were labeled with ^33^P-orthophosphate for 1 h, before treatment with 500 nM flg22. Lipids were extracted and separated by TLC in an acidic solvent system. PA content was normalized to structural phospholipids and the obtained values were normalized to mock controls. Statistical analysis was done by Kruskal–Wallis one-way ANOVA followed by post hoc pairwise comparisons. Data are presented as means ± se. Asterisks denote statistically significant difference between variants, *P* < 0.05, *n* = 9. C, Kinetics of ROS production in seedlings after a 500-nM flg22 treatment, given as relative luminescence units (luminol-peroxidase assay), data are presented as means ± se, *n* = 8–12. D, Callose deposition 24 h after flg22 treatment. Aniline blue staining and fluorescence microscopy were used for callose visualization. Each image is a composite image, scale bar = 500 µm (left panel); quantification of callose particles (per ROI), right panel. Results are displayed as boxplots (center line, median; box limits, upper and lower quartiles; whiskers, 1.5× interquartile range; and circles, individual values of independent samples). Different letters indicate statistically significant different values. One-way ANOVA, Tukey’s HSD, *P* < 0.05. At least 30 independent cotyledons were analyzed for each variant. E, Resistance to *P. syringae* pv. *tomato* DC3000 (*Pst) hrcC-*. Fourteen-day-old seedlings were flooded by a *Pst hrcC-* suspension (OD_600_ = 0.01) for 2 min, then the suspension was removed and after 2 days, the internal bacterial population was counted. Left panel, seedlings at 2 dpi; Right panel, infection development in plant tissues, log10 CFU/mg. Results are displayed as boxplots (center line, median; box limits, upper and lower quartiles; whiskers, 1.5× interquartile range; and circles, individual values of independent samples). Different letters indicate statistically significant different values (one-way ANOVA, Tukey’s HSD, *P* < 0.05, *n* = 18–23).

In *A. thaliana*, the DGK gene family contains seven members belonging to three clusters according to their domain structures and sequence similarities (Gómez-Merino et al., 2004; Escobar-Sepúlveda et al., 2017; Scholz et al., 2022). To identify the DGK isoforms contributing to flg22-triggered PA production, a screening of different knock-out mutant lines *dgk1*, *dgk4*, and *dgk5.1* that included members of all three DGK clusters was carried out. The mutant lines *dgk1* and *dgk4*, and WT seedlings showed a 30%–50% increase of PA content, 10 min after a 500-nM flg22 treatment (Supplemental Figure S3). On the other hand, the *dgk5.1* mutant line, carrying a T-DNA insertion in the At2g20900 locus (Figure 4A), showed a significantly impaired flg22-induced PA production (Figure 4B). Therefore, this DGK isoform appeared to be mainly responsible for PA formation in response to flg22. It should be noted that the *dgk5.1* line was a backcrossed line (see Materials and methods for details) and *DGK5* transcripts could not be detected by RT-qPCR (Supplemental Figure S4, for the list of primers used in this study see Supplemental Table S1).

To further analyze the role of the *dgk5.1* mutation in PTI, ROS production and callose deposition were followed as canonical examples of early and late responses to flg22, respectively. In both *dgk5.1* and WT plants, a classical ROS production response after PAMP recognition was observed. However, in the *dgk5.1* mutant, the maximum ROS level (as seen from the luminescence signal) was substantially lower than in WT plants, thus indicating the direct involvement of DGK5 in the observed process (Figure 4C). An important part of cellular reprogramming in response to flagellin recognition relies on a mitogen-activated protein kinase (MAPK) cascade (Asai et al., 2002). To place DGK5 within this context, a MAPK assay was performed on seedlings 1 h after the application of 500 nM flg22. A similar MAPK6 and MAPK3 activation was detected in both WT and *dgk5.1* plants (Supplemental Figure S5), suggesting that DGK5 was situated downstream from MAPK activation, or regulates an independent set of signaling events. Callose deposition was also measured in seedlings exposed to 500 nM flg22 for 24 h. In *dgk5.1* plants, flg22 induced a lower deposition of callose than in the WT (Figure 4D). Collectively, these results showed that DGK5 played a role in mediating flagellin responses but that it was not absolutely required.

### DGK5 plays a role in defense against *P. syringae*

To study whether the observed DGK5-dependent effects are required for efficient plant immunity, development of the infection process was followed in seedlings inoculated with the foliar pathogen *P. syringae* pv. *tomato* DC3000 (*Pst* DC3000). To separate distinct contributions of DGK5 to PTI and effector-triggered immunity in the context of the whole immune response, the *Pst* DC3000 *hrcC-* mutant strain was also used. This strain is deficient in the type III secretion system. As a positive control of depleted plant immunity, *NahG* plants, expressing bacterial salicylate hydroxylase that degrades SA to catechol (Delaney et al., 1994), were used. These plants are known to be impaired in SA-dependent immunity and allow a more intense bacterial growth in the apoplast (Delaney et al., 1994; Leontovyčová et al., 2019). At 2 dpi, the number of *Pst* DC3000 *hrcC-* bacteria in leaves was significantly higher in the *dgk5.1* mutant compared with WT plants, suggesting an enhanced susceptibility to pathogen attack when the DGK5 function was absent. The bacterial content in *dgk5.1* was comparable to that found in *NahG* seedlings (Figure 4E). Notably, when seedlings were infected with the native *Pst* DC3000 strain, the *dgk5.1* mutant response did not differ from that of the WT (Supplemental Figure S6). These results support the importance of DGK5 in the establishment of PTI.

### The immune-related phenotype is based on DGK5 activity

In an attempt to give further support to our observations, different T-DNA insertion lines of *dgk5* were tested in phenotype assays. However, *DGK5* transcript levels were found not to decrease in all of the tested alleles (Supplemental Figure S7, A–C). Accordingly, there was also no change in PTI-associated responses in these lines when compared with the WT (Supplemental Figure S7, D and E). To circumvent this, several stable transgenic lines were generated by transforming back-crossed *dgk5.1* plants with DGK5 driven by its native promoter with the aim of complementing the phenotype. The full genomic sequence of the *DGK5* gene was cloned either with its 3′-UTR end (pDGK5::DGK5-3′UTR; two independent lines denoted COM1 and COM2) or fused to GFP (pDGK5::DGK5-GFP; two independent lines denoted COM3 and COM4). To evaluate whether complementation of the *dgk5.1* phenotype was dependent on DGK5 enzymatic activity, a kinase-dead DGK5 line (pDGK5::DGK5^G112A^-GFP; line denoted MUT) was studied. To this end, the conserved Gly112 in the catalytic domain, which is important for enzymatic activity (Franks et al., 2017), was replaced by an Ala (G112A). The same strategy was recently used in plants for the DGK5 ortholog from tobacco (*Nicotiana tabacum*) NtDGK5 (Supplemental Figure S8 and Scholz et al., 2022). The level of *DGK5* transcripts was measured in seedlings of all transgenic lines by RT-qPCR and the presence of DGK5-GFP protein in all GFP-tagged lines was verified using anti-GFP antibodies (Supplemental Figure S9).

**Figure 5:**
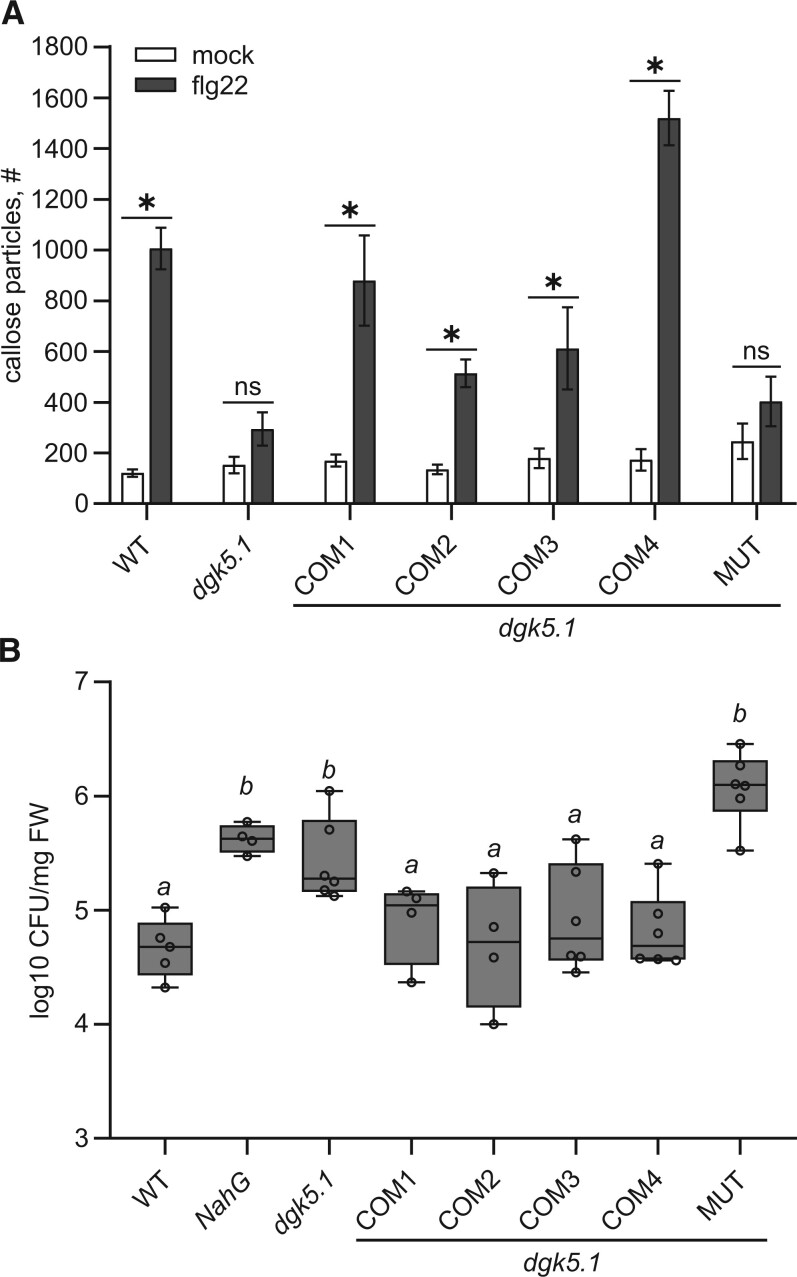
Expression of a functional DGK5 complements the PTI-related phenotype of the *dgk5.1* mutant. A, Callose deposition 24 h after flg22 treatment, quantification of callose particles (per ROI). Statistical analysis was done by Tukey’s one-way ANOVA followed by post hoc pairwise comparisons. Data are presented as means ± se. Asterisks denote statistically significant difference between variants, *P* < 0.05, *n* = 24–36; ns, not significant. B, Resistance to *P. syringae* pv. *tomato* DC3000 (*Pst*) *hrcC-*. Fourteen-day-old seedlings were flooded with a *Pst hrcC-* suspension (OD_600_ = 0.01) for 2 min, then the suspension was removed and the internal bacterial population was counted after 2 days, log10 CFU/mg. Results are displayed as boxplots (center line, median; box limits, upper and lower quartiles; whiskers, 1.5× interquartile range; and circles, individual values of independent samples). Different letters indicate statistically significant different values (one-way ANOVA, Tukey’s HSD, *P* < 0.05, *n* = 4–8). COM1-4 denote back-crossed *dgk5.1* lines complemented with pDGK5::DGK5-3 ′ UTR (two independent lines COM1 and COM2) or with pDGK5::DGK5-GFP (two independent lines COM3 and COM4). MUT denotes back-crossed *dgk5.1* line transformed with kinase-dead pDGK5::DGK5^G112A^-GFP.

To test PTI-associated responses, callose deposition upon flg22 treatment and resistance to *Pst* DC3000 *hrcC-* were chosen as the readout of phenotypes. In contrast to *dgk5.1* plants, all complementation lines accumulated significantly more callose spots when induced by flg22, reminiscent of the WT plant situation. On the other hand, the amount of callose spots remained at *dgk5.1* levels after flg22 treatment of the mutated catalytically inactive line (Figure 5A). Similarly, only *dgk5.1* and the inactive mutated DGK5 line (and *NahG* as a positive control) showed enhanced *Pst hrcC-* susceptibility, whereas all complementation lines mimicked the behavior of WT plants (Figure 5B). These results show that DGK5 enzymatic activity is crucial for the PTI-related response.

### DGK5 mediates basal expression of stress-responsive genes and regulates flg22-responsive genes

To better understand the role of DGK5 in plants, transcriptomic studies of WT and *dgk5.1* seedlings in control conditions and after short-term exposure to flg22 were performed. Eleven-day-old seedlings grown in liquid medium were treated with 1 µM flg22 for 60 min and subjected to CATMA (Complete Arabidopsis Transcriptome MicroArray) analyses. Three independent repetitions were carried out, with two dye-swaps for three comparisons: “WT versus *dgk5.1*”; “WT versus WT+flg22”; and “*dgk5.1* vs. *dgk5.1+*flg22” (Supplemental Figure S10 and Supplemental Table S2). Selected candidates from the microarray study were validated by RT-qPCR (Supplemental Figure S11).

We first focused on the “WT versus *dgk5.1*” comparison. In control conditions, 348 genes appeared to be down-regulated while 145 were up-regulated in the *dgk5.1* mutant compared with the WT. A Gene Ontology classification was performed (Supplemental Figure S12). Concerning the repressed genes, there was an over-representation of the categories “structural molecular activity” among the *Molecular function* classes and “ribosome” among the *Cellular component* classes. The categories “response to abiotic or biotic stress” and “response to stress” were overrepresented among the *Biological Process* categories, both for genes induced or repressed by the *dgk5.1* mutation (Supplemental Figure S12). A *Signature* analysis was performed using Genevestigator. This tool allowed to associate our signature—consisting of genes in our list and their corresponding expression level values as log2 ratio—to curated microarray experiments associated with “biotic,” “hormone,” “stress,” and “temperature” (423 experiments). In the top 10 most different experiments, six corresponded to response to biotic agents (Supplemental Figure S13). For instance, *GSTU4* (*GLUTATHIONE S-TRANSFERASE TAU 4*), *CYP82C2* (*CYTOCHROME P450*, *FAMILY 82*, *SUBFAMILY C*, *POLYPEPTIDE 2*), *MAPKKK19* (*MAPK KINASE KINASE 19*), and *EDS5* (*ENHANCED DISEASE SUSCEPTIBILITY 5*) are genes induced by biotic stress but repressed by the *dgk5.1* mutation. Our data thus indicated that the *dgk5.1* mutation led to a transcriptomic response that impaired the basal expression of genes associated with the response to biotic agents.

**Figure 6:**
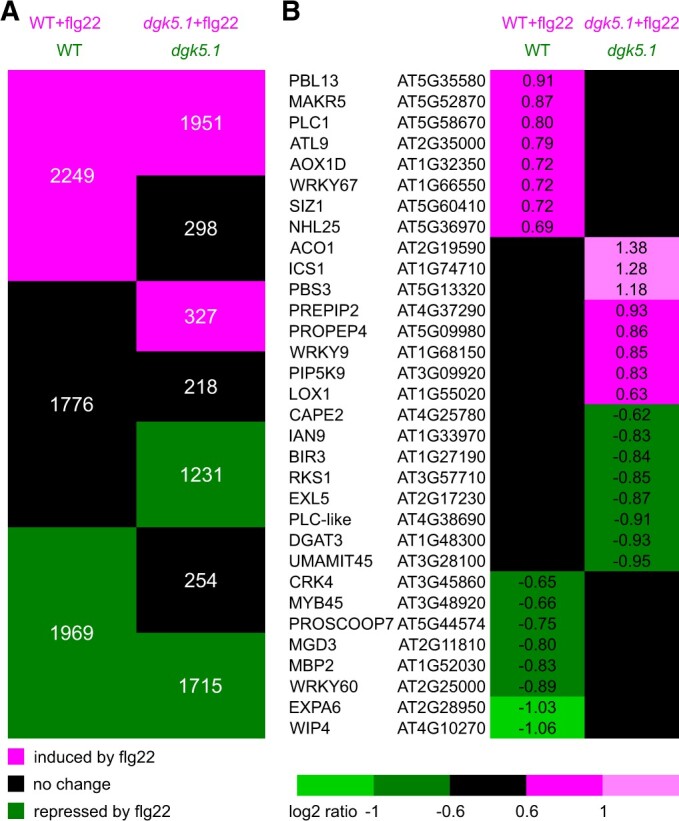
Comparison of a transcriptomic profile of the *dgk5.1* mutant versus the WT in response to flg22. Eleven-day-old seedlings were treated with 1 µM flg22 for 60 min and subjected to a transcriptome analysis. A, Genes can be clustered in categories depending on their expression in the following comparisons: “WT versus WT+flg22” and “*dgk5.1* versus *dgk5.1+*flg22.” B, Response of selected genes for which the *dgk5.1* background modified the “differentially expressed” status in response to flg22. Ratios are expressed as log2 of the transcript level in the condition written in magenta to that in the condition written in green. Magenta cells show induced genes in the condition written in magenta, while green cells indicate repressed genes in the condition written in magenta; black cells show no significant expression difference between the two conditions.

Concerning the response to flg22, 2249 and 1969 genes were induced and repressed, respectively, by flg22 in WT plants. In the d*gk5.1* mutant, 2278 and 2946 genes were induced and repressed, respectively, by flg22 (Figure 6A). One way to investigate DGK5-influenced gene responses to flg22 is to identify genes whose expression was altered enough in the *dgk5.1* mutant versus that in the WT so that its “response to flg22” assignation changed from “differentially regulated” to “non-differentially regulated” or, reversely, from “non-differentially regulated” to “differentially regulated.” Among the genes that were induced by flg22 in the WT, 298 appeared as non-differentially regulated in response to flg22 in the *dgk5.1* background. Among these genes was a receptor-like cytoplasmic kinase *PBL13* (*AvrPphB SUSCEPTIBLE1-LIKE1*), *PLC1*, *WRKY67* (*WRKY DNA-BINDING PROTEIN 67*) transcription factor, and *SIZ1* (*SALT INDUCED ZINC FINGER PROTEIN1*) (Figure 6B). Among the genes that were repressed by flg22 in the WT, 254 were non-differentially regulated in response to flg22 in the *dgk5.1* background including *MYB45* (*MYB DOMAIN PROTEIN 45*), *WRKY60*, and *PROSCOOP7* (*PRECURSOR OF SERINE-RICH ENDOGENOUS PEPTIDE 7*). On the other hand, among genes whose expression was not altered in the WT, 327 and 1231 were up-regulated or down-regulated, respectively, in the *dgk5.1* background (Figure 6A). Among the genes that were not differentially expressed in the WT but induced in the mutant were *ICS1* (*ISOCHORISMATE SYNTHASE 1*), *PBS3* (*AvrPphB SUSCEPTIBLE3*), *PROPEP4* (*ELICITOR PEPTIDE 4 PRECURSOR*), and *LOX1* (*LIPOXYGENASE 1*) (Figure 6B). Among the genes that were not differentially expressed in the WT but repressed in the mutant were *BIR3* (*BAK1-INTERACTING RECEPTOR-LIKE KINASE 3*), *EXL5* (*EXORDIUM-LIKE 5*), and *PLC-like* (Figure 6B). In total, 2110 (298 + 254 + 327 + 1231) genes were identified whose response to flg22 assignation changed in *dgk5.1* when compared with the WT background (Supplemental Table S2).

In this way, DGK5-dependent genes implicated in the response to flg22 were identified. The DGK5-dependent pathway appeared to have a dual action, enhancing the responses to flg22 for some genes (therefore no longer responsive in the mutant background) but also participating to fine-tune (repress) the response of other genes, which led to a response (induction or repression) in the mutant background.

### DGK5 protein binds membrane phospholipids in vitro and localizes to the PM

**Figure 7:**
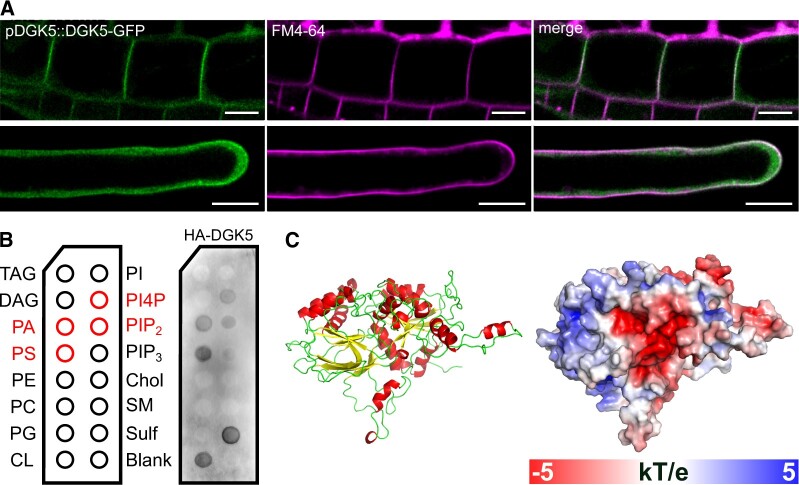
DGK5 subcellular localization, lipid-binding properties, and structural model. A, The localization of DGK5-GFP in the root transition zone (upper panels) and in root hairs (lower panels) of *dgk5.1* seedlings stably transformed with pDGK5::DGK5-GFP. FM4-64 dye (5 nM) was used to visualize PMs. Bars = 10 μm. B, Lipid-binding properties of DGK5 determined using a protein–lipid overlay assay (membrane lipid strip). HA-tagged DGK5 protein was prepared using the TNT SP6 High-Yield Wheat Germ in vitro coupled transcription/translation system and lipid-bound proteins were detected with an anti-HA antibody. Chol, cholesterol; CL, cardiolipin; PI4P, PI 4-phosphate; PIP_3_, PI 3,4,5-trisphosphate; PS, phosphatidylserine; SM, sphingomyelin; Sulf, 3-sulfogalactosylceramide; and TAG, triacylglycerol. C, The homology model of DGK5 (left panel) and the electrostatic potential (right panel) mapped onto the solvent accessible surface of the DGK5 model calculated using the APBS program.

To obtain an insight into the cellular localization of DGK5, the DGK5 coding sequence (CDS) was C-terminally or N-terminally fused to GFP, expressed under the control of the *35S* promoter in transiently transformed *N. benthamiana* leaf epidermis, and fusion protein localization was monitored by confocal microscopy. While both GFP-DGK5 and DGK5-GFP localized strongly to the PM, part of the fluorescence signal was found in the cytoplasm and interestingly, also in the nucleus (Supplemental Figure S14A). Next, Arabidopsis stable transgenic lines expressing GFP-tagged DGK5 under the control of its native promoter (pDGK5::DGK5-GFP), that complemented the *dgk5.1* mutant phenotype, were analyzed by following the GFP fluorescence signal in Arabidopsis roots. Despite a ubiquitous cytoplasmic signal, DGK5-GFP in the complemented lines also showed a PM localization, most prominently in the transition zone and in the apex of root hairs. The PM localization of DGK5 was also confirmed by its co-localization with the PM marker FM4-64 (Figure 7A).

A PM localization of DGK5 might be caused by its interaction with PM-specific lipid or protein partners. Therefore, the ability of DGK5 to interact with a spectrum of membrane lipids was tested using a protein–lipid overlay assay. HA-tagged DGK5 was prepared using an in vitro transcription/translation system and incubated with a membrane lipid strip. Interestingly, DGK5 possesses the ability to bind multiple anionic phospholipids in vitro, particularly those that are enriched in the PM (Figure 7B) such as phosphoinositides and PA. Since the DGK5 sequence did not contain any known phospholipid-binding domains or polybasic amino acid stretches, a 3D homology model of DGK5 was constructed and analyzed for putative membrane-binding regions. This suggested that DGK5 had a compact, globular, heart-shaped structure, corresponding to predictions for clade III DGK isoforms of tobacco (Scholz et al., 2022). The mapping of surface electrostatic potential onto the model revealed that DGK5 had a strong bipolar charge distribution with a negatively charged pocket surrounded by an area with positive surface charge (Figure 7C) that could be responsible for targeting to negatively charged PM lipids.

**Figure 8:**
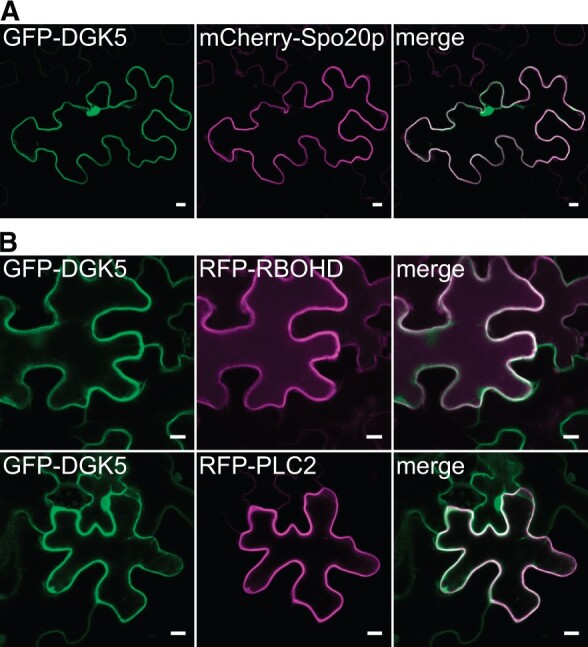
DGK5 localizes to the PM, like PA biosensor, RBOHD, and PLC2. *Nicotiana benthamiana* plants were co-transformed by equal amounts of *A. tumefaciens* carrying GFP-DGK5 and (A) the PA biosensor mCherry-NES-2xSpo20p-PABD or (B) RFP-RBOHD (upper panel) or RFP-PLC2 (bottom panel) constructs. NES, nuclear exporting signal; PABD, PA-binding domain; and RBOH, respiratory burst oxidase homolog. Bars = 10 μm.

Then an overlap in distribution of DGK5 and PA was tested in vivo using live-cell microscopy. To this end, a tandem variant of Spo20p-PABD with a nuclear exporting signal (mCherry-NES-2xSpo20p-PABD) was made, as this was necessary for the correct localization of this PA biosensor in plant vegetative tissues (Platre et al., 2018). This construct was co-transformed with GFP-DGK5 or DGK5-GFP constructs into *N. benthamiana* leaf epidermis. It was seen that the fluorescent signals of both DGK5-GFP-fusions clearly overlapped with that of the PA biosensor at the PM (Figure 8A and Supplemental Figure S14B).

Recently, D’Ambrosio et al. (2017) revealed that PLC2 affected PTI and was associated with RBOHD. As PLC activity produces DAG, a substrate of DGK, the possible interplay of DGK5 with these putative partners of the signaling cascade was studied. Constructs containing either RBOHD or PLC2 CDS N-terminally fused to RFP were co-transformed with GFP-DGK5 into *N. benthamiana* leaf epidermis. This confirmed that RBOHD and PLC2 localized to the PM, like GFP-DGK5 (Figure 8B). Collectively, this indicated that DGK5 can bind to the PM, thus suggesting its involvement in the interplay with other signaling partners during PTI.

## Discussion

As a component of plant stress response signaling, PA can be produced by different pathways, either by PLD hydrolyzing structural lipids like PC or by DGK phosphorylating DAG. The DAG, substrate of DGK, can result from the action of NPCs hydrolyzing PC (Pokotylo et al., 2013) or from PI-dependent PLC hydrolyzing PIP_2_ and/or PIP (Pokotylo et al., 2014). PA can be further phosphorylated to DAG pyrophosphate by PA kinase, or metabolized to lyso-PA through PLA_2_ activity (Testerink and Munnik, 2011; Escobar-Sepúlveda et al., 2017). By combining different approaches including radioactive labeling and the use of pharmacological agents, we have shown that flg22 activates a PI-PLC/DGK module that leads to PA accumulation (Figures 1 and 2). Our biochemical data were further corroborated by genetic and cell biology analyses that pinpointed DGK5 as the major isoform responsible for the production of flg22-induced PA.

Our data correspond with current knowledge of early stages of plant–microbe interactions. Hung et al. (2014) reported that Ca^2+^-release after elicitor treatment and the expression of some flg22-responsive genes were attenuated in plants deficient in InsP-5-ptase. InsP-5-ptase is an enzyme that participates in the turn-over of soluble inositol-phosphates originating from inositol-trisphosphate produced by PI-PLC. Inhibition of PI-PLC activity by pre-treatment of cotyledons of Arabidopsis seedlings using the PI-PLC inhibitor U73122 suppressed flg22-mediated endocytosis of the receptor-like kinase FLS2, and blocked immune responses. Furthermore, pre-treatment with U73122 also suppressed the proton influx induced by flg22 in tobacco suspension-cultured cells expressing the tomato (*Solanum lycopersicum*) Cf-4 protein (Abd-El-Haliem et al., 2016). In tobacco cells, a PI-PLC/DGK pathway was shown to be activated in response to the PAMP cryptogein, a 10-kDa protein secreted by the oomycete *Phytophthora cryptogea* (Cacas et al., 2017).

The majority of defense responses to PAMPs in plant cells are activated via specific receptor complexes. For flagellin, this cascade starts with the conserved peptide flg22 interacting with plant receptor kinase FLS2 (Gómez-Gómez and Boller, 2000; Chinchilla et al., 2006). BAK1 directly interacts with FLS2, acting as a co-receptor by recognizing the C terminus of flg22-bound FLS2 (Chinchilla et al., 2007; Heese et al., 2007; Sun et al., 2013). Receptor-like cytoplasmic kinase BIK1 is another component of the immune receptor complex. Using mutant seedlings, we established that flg22-triggered activation of the PI-PLC/DGK pathway was downstream of FLS2/BAK1 and BIK1. BIK1 has been reported to participate in a PAMP-induced increase of calcium levels (Li et al., 2014; Ranf et al., 2014; Monaghan et al., 2015), to positively regulate flg22-triggered calcium influx and to directly interact and phosphorylate the RBOHD at specific sites in a calcium-independent manner to enhance ROS generation (Ma et al., 2017). Furthermore, BIK1 and RBOHD were proposed to be part of a transduction pathway controlling stomatal movements in response to flg22 (Li et al., 2014). RBOHD was reported to mediate most of the ROS production during PTI (Nühse et al., 2007; Zhang et al., 2007). We show that PA accumulation in response to flg22 was not downstream of RBOHD (Figure 3). On the contrary, ROS accumulation was downstream of PA production since inhibiting PA accumulation (either by a pharmacological approach or by the use of the *dgk5.1* mutant) resulted in an attenuated ROS accumulation (Figures 2 and 4). Interestingly, RBOHD possesses a PA-binding domain and the binding of PA activates the enzyme during ABA-mediated ROS production and stomatal closure (Zhang et al., 2009). RBOHD also participates in the control of stomatal movements via SA, a process that also relies on PA (Kalachova et al., 2013). Recently, another PA-producing enzyme, Arabidopsis PLDδ, was implicated in the plant reaction to chitin, a fungal PAMP (Xing et al., 2019), suggesting the existence of non-redundant PA signaling pathways in plant immunity. Besides, in response to flg22, Arabidopsis PLDβs were shown to act upstream of ROS signaling to trigger actin remodeling through inhibition of CAPPING PROTEIN activity (Cao et al., 2022).

Callose accumulation is another plant response to pathogen attack. It can be regulated at the transcriptional and translational levels, and during enzyme transport via vesicular trafficking. Yet, its role in immunity is still unclear. Callose deposition had been associated with both non-host resistance and effector-triggered immunity. Interestingly, callose deposition was seen to be dependent of RBOHD (Luna et al., 2010). Using a back-crossed mutant T-DNA insertional line containing an inactive DGK5 enzyme compared with various complemented lines, we showed that flg22-triggered callose accumulation was dependent on DGK5 activity (Figures 4 and 5).

DGK activity has been reported in several plant species including tobacco, wheat (*Triticum aestivum*), tomato, Arabidopsis, rice (*Oryza sativa*), and apple (*Malus prunifolia*) (Escobar-Sepúlveda et al., 2017). Plant DGKs can be separated into three clusters (Gómez-Merino et al., 2004, 2005; Scholz et al., 2022). AtDGK1 and AtDGK2 are part of cluster I and they possess a hydrophobic segment required for membrane targeting (Vaultier et al., 2008). AtDGK3, AtDGK4, and AtDGK7 are members of cluster II while AtDGK5 and AtDGK6 have been assigned to cluster III. Our data suggest that AtDGK5 plays a role in the flg22 transduction pathway, upstream of RBOHD. Interestingly, Cacas et al. (2017) showed that the DGK upstream of RBOHD involved in the tobacco response to cryptogein was from cluster III. This points to a conserved function of type III-DGKs in response to PAMPs.

Based on transcriptomic analyses, gene sets whose expression levels were positively or negatively regulated by DGK5 activity in the Arabidopsis basal transcriptome have been discovered. Interestingly, there was a positive association between the genes regulated by basal DGK5 and the immune response as genes repressed in the *dgk5.1* mutant versus WT were mostly involved in stress responses, including biotic stresses (Supplemental Figure S13). Using a pharmacological approach, we had previously shown that DGK controlled the basal expression of a set of genes in Arabidopsis suspension cells (Djafi et al., 2013; Ruelland et al., 2014; Kalachova et al., 2015). The data presented in this current work confirmed the importance of the PI-PLC/DGK pathway in the maintenance of basal gene expression and pointed out that a basal DGK5 activity is required for the positive expression of a basal immune response. In response to flg22, gene sets whose response to this peptide was altered in the *dgk5.1* mutant were identified, thus confirming the importance of the DGK5-dependent pathway. Indeed, 298 genes induced by flg22 in WT seedlings were no longer induced in the *dgk5.1* mutant background, whereas 254 genes repressed by flg22 in WT seedlings were no longer repressed in the *dgk5.1* mutant background. In both cases, this represented only 13% of flg22-responsive genes in the WT. Since this did not represent a large effect on gene expression, we hypothesize that the role of DGK5 is more in terms of fine-tuning gene expression. This is corroborated by the fact that some genes not detected as flg22-responsive in WT seedlings were deregulated in the mutant background.

Therefore, it is clear that DGK5 is involved, at least partially, in the regulation of flg22-induced ROS production, callose accumulation, and gene expression. This is associated with DGK5-dependent PA production that acts downstream from FLS2/BAK1 and BIK1. As a result, the *dgk5.1* mutant is compromised in the establishment of pathogen resistance. It appears that both PI-PLC and DGK are part of plant primary responses to flg22. Interestingly, the phenotypes of *PLC2-*silenced plants (D’Ambrosio et al., 2017) are similar to those observed for *dgk5.1* mutant plants. Both are more susceptible to the type III secretion system-deficient bacterial strain *P. syringae* pv. *tomato* (*Pst*) DC3000 *hrcC-* but not to *Pst* DC3000. Furthermore, both mutants displayed reduced ROS production and callose deposition after flg22 treatment whereas they maintained WT levels of MAPK activation and *FRK1* expression. Moreover, it was found that PLC2 is associated with RBOHD. Here, we confirmed that RBOHD, PLC2, and DGK5 localize at the PM (Figure 8). When taken together, this suggests an interplay between DGK5 and PLC2 during PTI.

Although DGK5 does not contain any known phospholipid-binding domains or polybasic stretches, we were able to show, using a 3D homology model of DGK5, an area with positive surface charge that could be responsible for targeting DGK5 to negatively charged PM lipids, such as phosphoinositides, PA, and phosphatidylserine (PS) as revealed by lipid–protein overlay assays (Figure 7). The PM distribution of these lipids is under tight control resulting in a locally modulated membrane charge (Platre et al., 2018), enabling the recruitment of proteins and thus contributing to the organization of cell membrane processes. Minor PM lipids such as phosphoinositides (Noack and Jaillais, 2017) and PA (Pokotylo et al., 2018) are often responsible for the recruitment of proteins to the plant PM although some studies have also described a role for PS in these protein–lipid interactions (Platre et al., 2019; Zhou et al., 2020).

**Figure 9:**
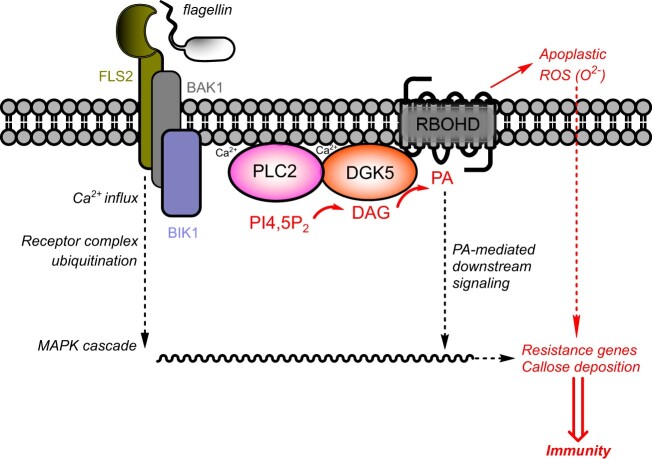
Simplified schematic model of lipid signaling associated with the flagellin signal perception cascade in Arabidopsis cells. The recognition of flg22 by the FLS2–BAK1–BIK1 complex initiates PI4,5P_2_ hydrolysis by PLC2 to form DAG and IP_3_. DAG is used as a substrate for DGK5 thus leading to PA formation. Newly produced PA then acts as a regulator of RBOHD-dependent ROS production, callose deposition, and gene expression thus resulting in resistance to bacterial pathogens. PI4,5P_2_, PI 4,5-bisphosphate.

Based on the presented work, the following working model is proposed (Figure 9). The PAMP flg22 activates the FLS2/BAK1 co-receptor that activates BIK1. As a result, a Ca^2+^ influx occurs that activates PI-PLC. Note that other regulating events of PI-PLC could be required, such as protein phosphorylation (Pokotylo et al., 2014). PI-PLC activity will produce DAG that is phosphorylated to PA by DGK5. Whether DGK5 activity is also subject to regulation, for instance by protein phosphorylation, needs more investigation. DGK5-associated PA can bind to and activate RBOHD leading to ROS production, required for callose accumulation. It is probable that a set of flg22-responsive genes also depends on RBOHD activation, but we cannot rule out a gene set independent of this. It is not yet established whether PLC and DGK5 form a complex that tunnels PA toward RBOHD.

## Conclusion

An updated model of signaling pathway after flagellin perception in plant cells is proposed, that incorporates the role of membrane phospholipids and their turnover regulated by a PI-PLC/DGK5 module. This pathway is a component of PTI that plays an important role in defense responses.

## Materials and methods

### Plant material

Arabidopsis (*A. thaliana*) ecotype Columbia-0 was used as the WT. Mutant lines were obtained from the Nottingham Arabidopsis Stock Centre (NASC): *fls2* (SALK_026801C), *bak1-4* (SALK_116202), *rbohD* (SALK_074825C), *bik1* (SALK_005291), *dgk1.1* (SALK_053412), *dgk4.1* (SALK_069158), *dgk5.1* (SAIL_1212_E10) (homozygous F2 from a back-crossed line), and *NahG* (Delaney et al., 1994).

Seedlings were grown in vitro in liquid medium or on agar-containing medium. Seeds were surface sterilized by hypochlorite:ethanol (1:4, v/v) solution and cultivated in 24-well (for phospholipid assay and callose measurement) or 96-well plates (for ROS assay) containing half-strength Murashige–Skoog medium supplied with vitamins and 5% (w/v) sucrose, pH 5.7 at 22°C under a 14-h/10-h light cycle. After 7 days of cultivation, old media were changed with fresh media. For *P. syringae* assays, plants were grown on MS/2 medium supplied with vitamins and 5% (w/v) sucrose, pH 5.7, 8 g L^−1^ agar on Petri plates. Plates were placed under a 14-h/10-h light cycle at 22°C.

Arabidopsis Col-0 suspension cells were cultivated as in Kalachova et al. (2016). Suspension cultures were sub-cultivated every 7th day and treatments described below were performed 7 days after sub-culturing.

*Nicotiana benthamiana* plants were cultivated in a greenhouse at 26°C under a 16-h/8-h light cycle, for up to 4–5 weeks.

### Pharmacological treatments

Peptide flg22 derived from the flagellin N-terminus of *Pseudomonas* sp. (QRLSTGSRINSAKDDAAGLQIA, Tebu-Bio, France) was diluted to a 0.2-mM stock solution in water. PI-PLC inhibitor U73122 and its inactive analog U73433 were diluted in DMSO:tert-butanol (1:2, v/v) to give a 10-mM stock solution. DGK inhibitor I (R59022) was dissolved in tert-butanol at a stock concentration of 10 mM. Wortmannin was dissolved in methanol at a stock concentration of 6 mM. As controls, cells were treated with the respective *solvent*. All studied inhibitors were added 30 min prior to flg22 treatment.

### Phospholipid labeling and lipid extraction

Seven-day-old suspension cells were labeled with 37 MBq L^−1 33^P-orthophosphate per 7 mL during the specified time according to a procedure previously described in Krinke et al. (2007). Lipids were extracted as described in Ruelland et al. (2002). For labeling of 14-day-old seedlings grown in liquid medium, cultivation media was replaced by 100 mM Tris–HCl, pH 6.15. Seedlings were equilibrated in this buffer for 2 h (400 μL per well), at 22°C, under light, prior to the addition of ^33^P-orthophosphate at 4 MBq [^33^P] per well. After a 1-h incubation, the reaction was stopped by placing seedlings from three wells (approx. 10–12 seedlings) into a Potter’s homogenizer, adding 4 mL of hot isopropanol and grinding after adding 3 volumes of chloroform:methanol:37% (v/v) HCl (50:100:1.5, v/v). The mixture was transferred to tubes and a two-phase system was induced by the addition of 1.25 volumes of chloroform and 1.25 volumes of 9% (w/v) NaCl in water. Tubes were vigorously shaken and left at 4°C overnight for two-phase formation. The lower organic phase was evaporated under a nitrogen stream. Lipids from suspension cells or seedlings were dissolved in 100 μL chloroform and stored at −20°C until further use.

### TLC and phosphor imaging

Lipids were analyzed after TLC (on 200 × 200 × 0.25 mm plates, Merck, Germany). Structural phospholipids and PA were separated in an acid solvent system composed of chloroform:acetone:acetic acid:methanol:water (10:4:2:2:1 [v/v/v/v]) (Lepage, 1967). Phosphoinositides were separated in an alkaline solvent system composed of chloroform:methanol:ammonia solution (5% [w/v]; 9:7:2 [v/v/v]). TLC plates were soaked in potassium oxalate solution before heat activation (Munnik et al., 1994). Lipids were spotted by an ATS4 automatic sampler (CAMAG). Radiolabeled spots were quantified by autoradiography using a Storm Phosphorimager (Amersham Biosciences). Separated phospholipids were identified by co-migration with authentic non-labeled standards visualized by primuline staining (under UV light) and verified according to a previously published protocol (Munnik et al., 1996).

### RNA extraction

Total RNA from cells was extracted using an RNeasy Plant Mini Kit (Qiagen, France). The quantity of extracted RNA was quantified from absorption values obtained using a NanoDrop spectrophotometer. RNA quality was assessed on 2.5% (w/v) agarose gels with 0.3% (v/v) GelRed staining.

### Transcriptome analysis

The transcriptome response to flg22 was monitored by a microarray analysis carried out at the Institute of Plant Sciences Paris-Saclay (IPS2, Orsay, France), using CATMAv5 arrays containing 31,776 gene-specific tags corresponding to 22,089 Arabidopsis genes (Crowe et al., 2003; Hilson et al., 2004). Two independent biological replicates were produced. For each biological repetition and each sampling point, samples were obtained by pooling RNA from three independent experiments. RNA was prepared as described in Krinke et al. (2009). Flg22 treatment was compared with a mock control and WT response was compared with that of the *dgk5.1* mutant. One technical replicate with fluorochrome reversal was performed for each biological replicate (i.e. four hybridizations per comparison). The labeling of cRNA with either Cy3-dUTP or Cy5-dUTP (Perkin-Elmer-NEN Life Science Products) and the hybridization to slides and scanning were performed as described in Lurin et al. (2004). Statistical analysis of the microarray data was performed as described in Kalachova et al. (2016).

Microarray data have been submitted in the international repository GEO (Gene Expression Omnibus, [Edgar et al., 2002], http://www.ncbi.nlm.nih.gov/geo, ProjetID GSE107745). All steps of the experiment, from growth conditions to bioinformatic analyses, were detailed in the CATdb database (Gagnot et al., 2008), http://tools.ips2.u-psud.fr/cgi-bin/projects/CATdb/consult_project.pl?project_id=455; ProjectID AU17-01_Dgk5.1_bis) according to the “Minimum Information About a Microarray Experiment” standards.

### In silico analysis of microarray data

Genes were classified using the Classification SuperViewer Tool developed by Provart and Zhu (2003). The classification source was set to Gene Ontology categories as defined by Ashburner et al. (2000). The frequency of a category was normalized to that of the whole Arabidopsis set. The mean and standard deviation for 100 bootstraps of our input set were calculated to provide some idea as to over- or under-representation reliability. Similarity analysis was performed using tools developed by Genevestigator (Hruz et al., 2008).

### Transcript abundance evaluation by RT-qPCR

cDNA was synthesized by reverse transcription of 4 μg of total RNA using an oligo-dT primer and Superscript III kit (Invitrogen). Real-time PCR was performed in a Step One Plus system (Applied Biosystems) using the Power SYBR Green PCR mastermix (Applied Biosystems). The amplification program consisted of an initial denaturation at 95°C for 10 min, 35 cycles of amplification at 95°C for 20 s, followed by 30 s at a Tm specific for each primer couple and 45 s at 72°C. *TIP41* (*TAP42 INTERACTING PROTEIN OF 41 kDa*, At4g34270) was used as reference gene. A log2 normalization was applied to the average transcript abundance level to obtain the same data range as in the transformed microarray data. Primers are listed in Supplemental Table S1.

### ROS production assay

Seven-day-old Arabidopsis Col-0 suspension cells were washed and equilibrated for 3 h under agitation in Erlenmeyer flasks in assay buffer (10 mM Tris–HCl, 175 mM mannitol, pH 7.2). Cells were then transferred to a 96-well black plate (175 µL of cells per well) and labeled with 5.8 M H2DCFDA for 15 min. Flg22 was then added by a dispenser in the microplate reader (Tecan). Fluorescence was measured for 6 h every 2 min, the excitation wavelength was set to 480 nm, and emission wavelength was 528 nm.

For assays with seedlings, Arabidopsis plants were cultivated for 10 days in liquid MS media supplemented with 0.5% sucrose and vitamins (pH 5.7), in a 96-well plate (1 seedling per well). Cultivation media was replaced by 200 µL of assay solution (50 mM Tris–HCl, pH 8.5, buffer solution containing 70 µg mL^−1^ luminol [Sigma-Aldrich], 40 µg mL^−1^ horseradish peroxidase and 100 nM flg22). Relative luminescence was measured during a 1-h period at 2 min intervals. Every well was measured separately and data from 12 independent measurements were used in one set of experiments.

### Callose deposition evaluation

Ten- to twelve-day-old seedlings grown in liquid medium (24-well plate, four to five seeds per well) were exposed to 1 µM flg22 for 24 h. Callose deposition was observed after aniline blue staining as in Kalachova et al. (2019). Cotyledons were imaged on Zeiss AxioImager microscope with ApoTome2, using EC Plan-Neofluar 5×/0.16 M27 and 10×/0.3 M27 objectives, upon UV fluorescence. Callose deposition was evaluated by ImageJ software, at least 12 independent cotyledons were analyzed per variant. For the analysis, one circular region of interest (ROI, diameter = 2 mm) mask was created and applied for all cotyledons. The images were thresholded and the background was subtracted. Callose deposition was evaluated as the number of particles (circularity index 0.5–1) and % of area with aniline blue fluorescence signal per ROI.

### Resistance to bacteria

Resistance to *P. syringae* was assessed on 14-day-old seedlings according to Ishiga et al. (2011).

### Molecular cloning

To create stable Arabidopsis transgenic lines, a DNA fragment starting 3,000 bp upstream of the Arabidopsis *DGK5* gene start codon to the end of its 3′UTR (pDGK5::DGK5-3′UTR; independent lines COM1 and COM2) was amplified from Arabidopsis Col-0 genomic DNA using specific primers PC1 and PC2 (Supplemental Table S1) and Q5 polymerase (New England Biolabs), then cloned into the pENTR3C vector (Invitrogen) via SalI and NotI restriction sites. This entry clone was recombined into the Gateway binary vector pGWB501 (Nakagawa et al., 2007b) by LR Clonase II (Invitrogen). To create GFP-tagged transgenic lines (pDGK5::DGK5-GFP; independent lines COM3 and COM4), a DNA fragment was amplified from Arabidopsis Col-0 genomic DNA using specific primers PC1 and PC3 and cloned into pENTR3C vector via SalI and NotI restriction sites. To create the GFP-tagged DGK5^G112A^ mutated line (pDGK5::DGK5^G112A^-GFP; line MUT), megaprimer MP-G112A-R was generated first using PC4 and PC5 primers and the WT DGK5 entry clone (see above) as template. Next, PC6 and MP-G112A-R were used as primers to produce part of pDGK5::DGK5^G112A^ sequence. The mutation-containing DNA was cut with BstBI and NdeI and used to replace the corresponding part in the WT sequence cloned in pENTR3C. Both entry clones for the GFP-tagged lines were recombined into the Gateway binary vector pGWB4 (Nakagawa et al., 2007).

To prepare constructs for transient expression, *DGK5*, *PLC2*, and *RBOHD* CDSs were amplified from Arabidopsis Col-0 cDNA using Q5 DNA polymerase. For transient expression in *N. benthamiana* leaves, PCR-amplified products flanked with KpnI/SalI and NotI sites (using primers PC3, PC7-PC12) were cloned into the pENTR3C entry vector (Invitrogen) and LR-recombined to the Gateway destination vector pGWB5, pGWB6 (Nakagawa et al., 2007), and pH7WGR2 (Karimi et al., 2002) for C-terminal GFP, N-terminal GFP, and N-terminal RFP fusions, respectively. To prepare the PA biosensor construct (pUBQ::mCherry:NES-2xSpo20p-PABD), NES-Spo20p-PABD was first amplified using PC13 and PC14 primers and YFP:Spo20p-PABD (Potocký et al., 2014) as template and introduced into mRFP:2xSpo20-PABD (Pejchar et al., 2020) using XbaI/SpeI sites to make mRFP:NES-2xSpo20-PABD. Then, NES-2xSpo20-PABD was cut with XbaI and SacI and cloned together with the ubiquitin promoter and mCherry into the binary vector pHD71 (kindly provided by Dr. Benedikt Kost).

The construct for in vitro transcription/translation (HA-DGK5) was made as follows: First, the *DGK5* CDS was amplified using specific primers PC15 (carrying an overhang for primer PC16) and PC8 from DGK5 in the entry vector as template. In a second step, a forward primer containing an added SalI site, Kozak sequence and HA-tag (PC16), the same reverse primer PC8, and the DGK5 product from the first step as template were used for PCR amplification. The final product was introduced into vector pTNT (Promega) via SalI and NotI restriction sites.

### Production of stable Arabidopsis transformant lines

Final constructs were transferred into *Agrobacterium tumefaciens* strain GV3101, which was used to transform back-crossed *dgk5.1* plants by the floral dip method (Clough and Bent, 1998). Transformants were selected on hygromycin plates.

### Transient expression in *N. benthamiana*

Fusion constructs, pUBQ::mCherry-NES-2xSpo20-PABD, p35S::GFP-DGK5, p35S::RFP-PLC2, and p35S::RFP-RBOHD were introduced into *A. tumefaciens* strain GV3101. Transient transformation was performed according to Li (2011). Samples were observed 24–48 h after transformation.

### Confocal microscopy

Fluorescence signals were visualized and captured using a Zeiss LSM 880 confocal microscope with the 63× objective. Following settings were used for GFP (laser, 488 nm; intensity, 2%; collection bandwidth, 489–552 nm; and gain, 700–850), FM4-64 (laser, 561 nm; intensity, 5%; collection bandwidth, 663–735 nm; and gain, 600–800), mCherry (laser, 561 nm; intensity, 2.2%; collection bandwidth, 590–666 nm; and gain, 800), and RFP (laser, 561 nm; intensity, 2%; collection bandwidth, 561–617 nm; and gain, 700) fluorescence.

### Homology modeling

DGK5 homology model was produced by Robetta (Kim et al., 2004), evaluated with Prosa (prosa.services.came.sbg.ac.at/prosa.php), WhatIf (swift.cmbi.ru.nl/servers/html/index.html), and PSVS (psvs.nesg.org/) servers. Electrostatic potentials were calculated using the package APBS (Baker et al., 2001). Blue, red, and white colors represent positive, negative, and neutral potentials, respectively. Pymol (www.pymol.org/) was used to visualize the structures.

### Protein–lipid overlay assay

Protein–lipid overlay assays were performed using N-terminal HA-tagged DGK5 and lipid strips (Echelon Biosciences). The HA-DGK5 construct was used as a template for in vitro coupled transcription/translation using the TNT SP6 High-Yield Wheat Germ Protein Expression System (Promega) in a total volume of 50 μL. The reaction product was used for the protein–lipid overlay assay as described previously (Kubátová et al., 2019).

### MAPK assay and Western blot

Seven-day-old Arabidopsis seedlings were collected (approximately 15), frozen in liquid nitrogen, and homogenized. Protein extraction and the MAPK assay protocol were carried out as in Fernandez et al. (2020). The presence of DGK5-GFP fusion protein in stable transgenic lines was shown by Western blotting and an anti-GFP antibody (dilution 1:2000, Agrisera AS15 2987).

### Data analysis and statistics

At least three biological repetitions were performed for all experiments, except for transcriptome array analysis. Mean values, standard errors of means, and the signiﬁcance of differences between samples were calculated using Excel, GraphPad Prism 8, and R software; *t*-test and ANOVA statistical analyses were applied. The exact statistical treatment and number of replicates are specified in the figure legends.

## Accession numbers

Sequence data from this article can be found in the Arabidopsis Genome Initiative under accession numbers: BAK1, AT4G33430; BIK1, AT2G39660; DGK1, AT5G07920; DGK4, AT5G57690; DGK5, AT2G20900; FLS2, AT5G46330; MAPK3, AT3G45640; MAPK6, AT2G43790; PLC2, AT3G08510; and RBOHD, AT5G47910.

## Supplemental data

**Supplemental Figure S1.** Accumulation of PA in response to flg22 in cell cultures with different times of labeling with ^33^P-orthophosphate.

**Supplemental Figure S2.** PA accumulation in Arabidopsis seedlings after flg22 treatment.

**Supplemental Figure S3.** Involvement of different DGKs in PA accumulation after flg22 treatment.

**Supplemental Figure S4.** Characterization of the *dgk5.1* mutant.

**Supplemental Figure S5.** MAPK phosphorylation is induced by flg22 treatment in WT and *dgk5.1*.

**Supplemental Figure S6.** Resistance to *P. syringae* pv. *tomato* (*Pst*) DC3000 did not differ between *dgk5.1* mutant and WT plants.

**Supplemental Figure S7.** *DGK5* T-DNA insertion lines that do not show the *dgk5.1* phenotype.

**Supplemental Figure S8.** Alignment of the DGK catalytic domain of DGK5 from Arabidopsis and tobacco.

**Supplemental Figure S9.** Transcription of the *DGK5* gene and presence of fused proteins in tested independent complementation lines of *A. thaliana*.

**Supplemental Figure S10.** Gene clustering according to transcriptomic data.

**Supplemental Figure S11.** Transcriptome validation.

**Supplemental Figure S12.** Enrichment in GO categories in gene sets induced or repressed in *dgk5.1* versus WT.

**Supplemental Figure S13.** Dissimilarity between the *dgk5.1*-responsive transcriptome and public transcriptome data.

**Supplemental Figure S14.** DGK5 localization in *N. benthamiana*.

**Supplemental Table S1.** List of primers used in this study.

**Supplemental Table S2.** Gene expression in *dgk5.1* and WT plants treated or not treated with flg22.

## Supplementary Material

kiac354_Supplementary_Data

## Notes

These authors contributed equally as first authors (T.K. and E.Š.)

T.K., P.P., and E.R. designed the experiments and research plans, and wrote the article with contributions from all authors. T.K., E.R., and N.D. performed the experiments with radiolabeling. T.K., E.R., S.P., and L.S.-T. performed the transcriptome analysis. N.D. and S.C. performed the first mutant characterizations. P.P., J.S., M.P., E.Š., and T.K. performed the cloning, protein characterization, and transient expression assays. E.Š. and T.K. performed the transformation and characterization of complementation lines. T.K. and L.B. performed the immunity-related experiments. P.P., L.B., and E.R. were involved in funding acquisition, resources, and project administration.

The author responsible for distribution of materials integral to the findings presented in this article in accordance with the policy described in the Instructions for Authors (https://academic.oup.com/plphys/pages/general-instructions) is: Přemysl Pejchar (pejchar@ueb.cas.cz).
