# Supplementary material for: DIACYLGLYCEROL KINASE 5 participates in flagellin-induced signaling in Arabidopsis
Source: Plant Physiol. 2022 Jul 28;190(3):1978–96. doi: 10.1093/plphys/kiac354 (PMC9614507; doi:10.1093/plphys/kiac354)
Supplement: kiac354_Supplementary_Data [file kiac354_supplementary_data.zip › Supplemental Material_PROD.pdf]

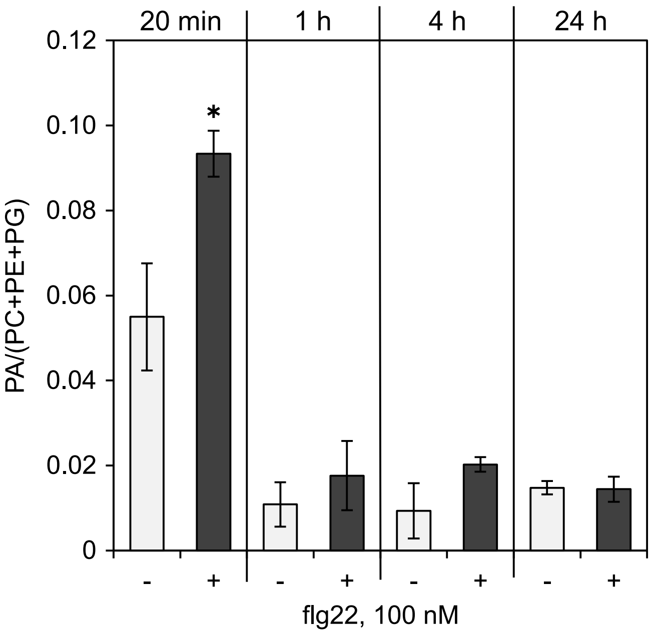

Supplemental Figure S1. Accumulation of PA in response to flg22 in cell cultures with different times of labeling with ^{33}P-orthophosphate. Seven-day-old suspension cells were labeled with ^{33}P-orthophosphate for different times (20 min, 1 h, 4 h, 24 h) and treated with flg22 for 10 min before lipid extraction. The radioactivity associated with PA was normalized to structural phospholipids (phosphatidyletanolamine (PE), phosphatidylcholine (PC), phosphatidylglycerol (PG)). Data are presented as means ± SD. Asterisk indicates sample that is different from control, $p<0.05$, unpaired *t*-test, n=3. PA, phosphatidic acid.

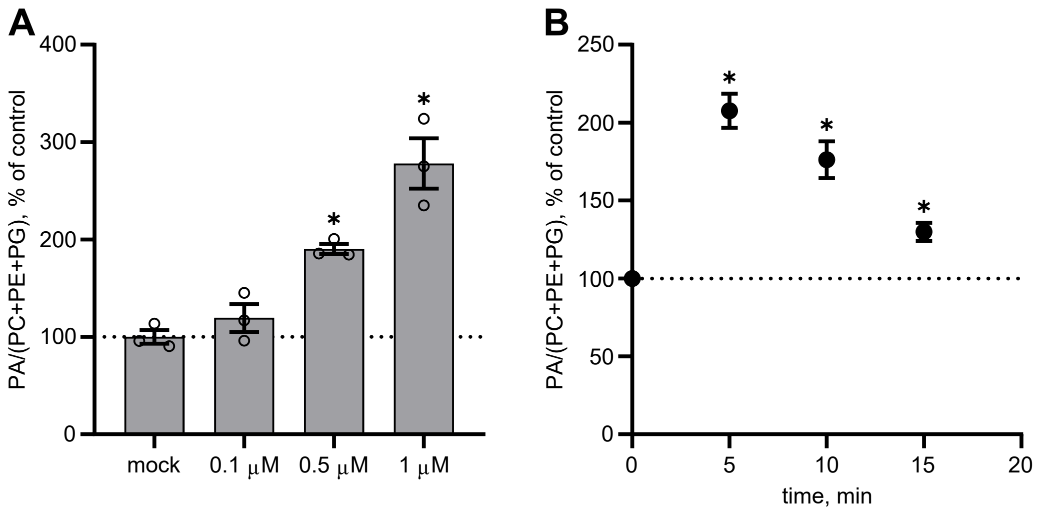

Supplemental Figure S2. PA accumulation in Arabidopsis seedlings after flg22 treatment. A, Dose response, 10 min of flg22 treatment. B, Kinetics of relative PA accumulation, 0.5 µM flg22 treatment. Seedlings were labeled with ^{33}P-orthophosphate for 1 h, treated with flg22 and then lipids were extracted and separated in an acidic solvent system (chloroform:acetone:acetic acid:methanol:water (10:4:2:2:1 [v/v/v/v]). PA content was normalized to structural phospholipids (phosphatidyletanolamine (PE), phosphatidylcholine (PC), phosphatidylglycerol (PG)), and the obtained values were normalized to mock controls. Data are presented as means ± SE. Asterisks indicate samples that are different from controls, $p<0.05$, paired *t*-test, n=3-4. The dashed line corresponds to the mean of control values. PA, phosphatidic acid.

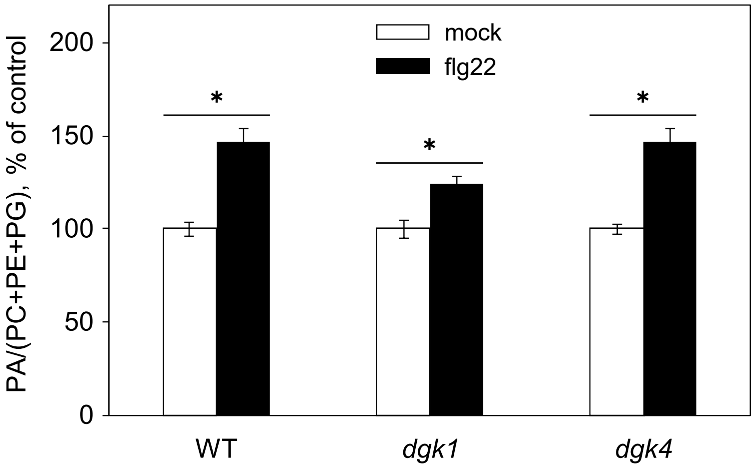

Supplemental Figure S3. Involvement of different DGKs in PA accumulation after flg22 treatment. Eleven-day-old seedlings were labeled with ^{33}P-orthophosphate for 1 h, treated with 500 nM flg22, and then lipids were extracted and separated in an acidic solvent system (chloroform:acetone:acetic acid:methanol:water (10:4:2:2:1 [v/v/v/v]). PA content was normalized to structural phospholipids (phosphatidylethanolamine (PE), phosphatidylcholine (PC), phosphatidylglycerol (PG)), and the obtained values were normalized to mock controls. Statistical analysis was done by Kruskal–Wallis one-way ANOVA followed by *post-hoc* pairwise comparisons. Data are presented as means ± SE. Asterisks denote statistically significant difference between variants, $p<0.05$, n=6-12. DGK, diacylglycerol kinase. PA, phosphatidic acid.

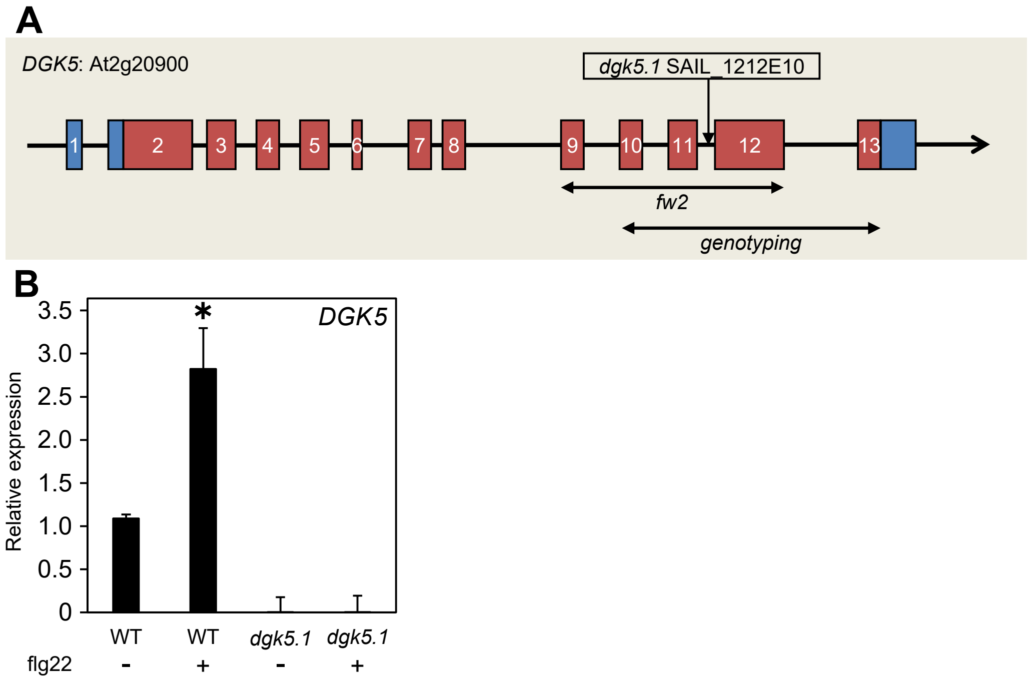

Supplemental Figure S4. Characterization of the *dgk5.1* mutant. A, Gene map and positions of the T-DNA insertion and primers used for genotyping and transcript evaluation. B, Transcription of DGK5 in the WT and the *dgk5.1* mutant after a 500 nM flg22 treatment. Data are presented as means ± SE. Asterisk denotes sample significantly different from control, $p<0.05$, n=3-4, unpaired *t*-test. DGK, diacylglycerol kinase.

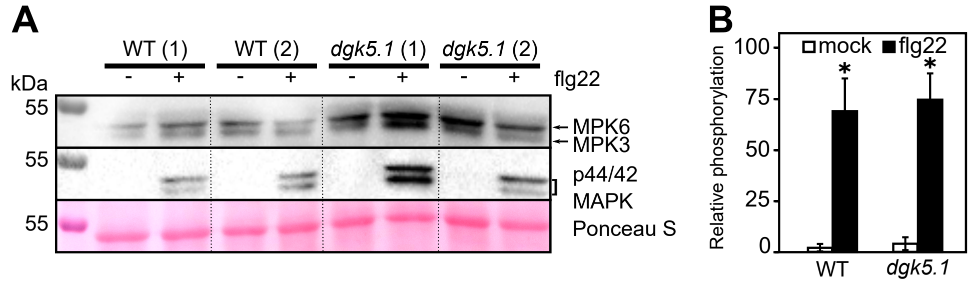

Supplemental Figure S5. MAPK phosphorylation is induced by flg22 treatment in WT and *dgk5.1*. A, Protein extracts were isolated from control and 1h flg22-treated seedlings. Western blots were first probed with an anti-phospho-44/42 MAPK antibody, and then stripped and an anti-MPK6/MPK3 antibody was used. Ponceau staining of Rubisco was used to control protein loading. Numbers in brackets stand for sample replicas. B, Comparison of relative phosphorylation of MPK6/MPK3 between treatments and tested lines. Data are presented as means ± SE. Asterisks denote samples significantly different from controls, p<0.05, n=4, unpaired *t*-test. n=4. DGK, diacylglycerol kinase; MAPK/MPK, mitogen-activated protein kinase.

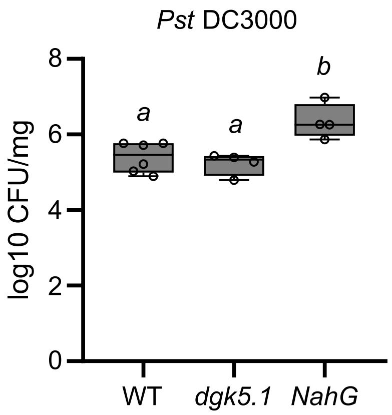

Supplemental Figure S6. Resistance to *P. syringae* pv. *tomato* (*Pst*) DC3000 did not differ between *dgk5.1* mutant and WT plants. 14-day-old seedlings were flooded with a *Pst* DC3000 suspension (OD_{600}=0.01) for 2 min, then the suspension was removed and after 3 dpi the internal bacterial population was counted as log10 CFU/mg. Results are displayed as boxplots (center line, median; box limits, upper and lower quartiles; whiskers, 1.5x interquartile range; circles, individual values of independent samples). Different letters indicate statistically significant different values (one-way ANOVA, Tukey's HSD, $p<0.05$, n=4-6).

A

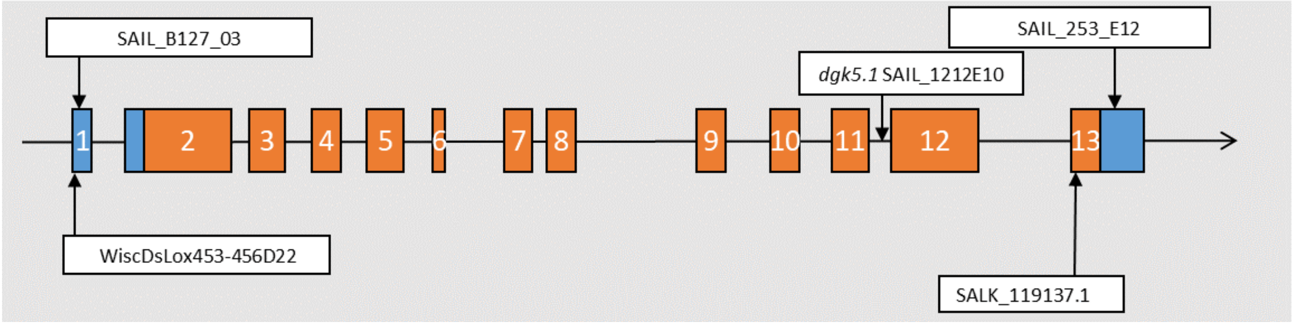

B

Name	Origin
dgk5.1	back-crossed line Sail_1212_E10
Sail_1212_E10	original Sail line, N863002
Sail_253_E12	original Sail line, *dgk5.2*
Sail_B127_03	original Sail line
Salk_119137.1	original Salk line, N869745
WiscDcLox453-456D22	original WiscDsLox line, N864675

C

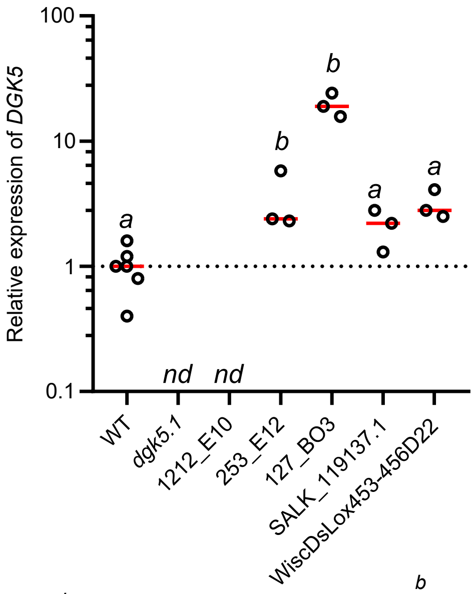

D

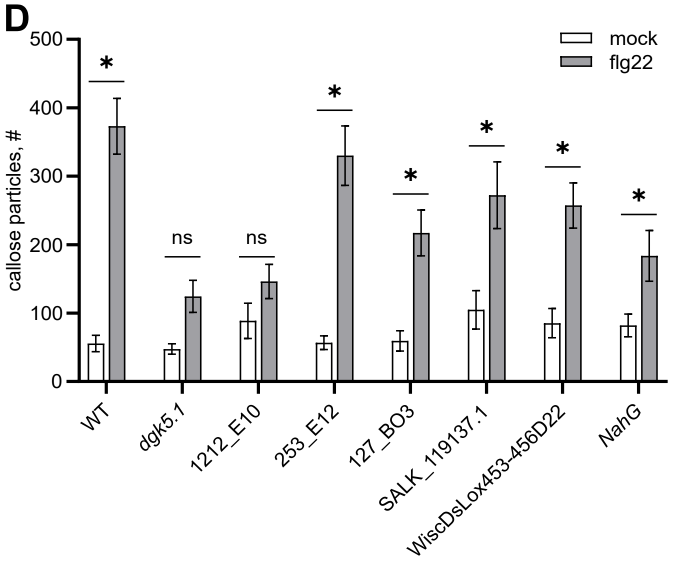

E

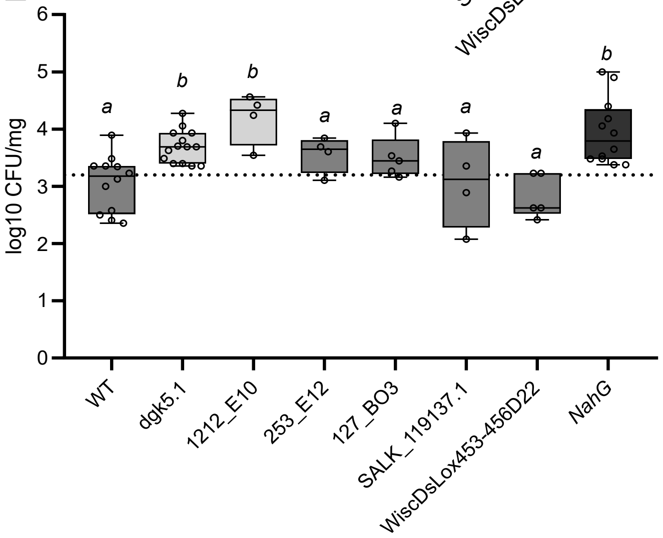

Supplemental Figure S7. *DGK5* T-DNA insertion lines that do not show the *dgk5.1* phenotype. A, Schematic representation of *DGK5* gene organization and the location of T-DNA insertions. B, Origin of the tested lines. C, *DGK5* transcription levels in tested lines. Red line indicates mean, circles represent individual values of independent samples. Different letters indicate statistically significant different values (one-way ANOVA, Tukey's HSD, $p<0.05$, n=3-6); nd, not detected. D, Callose deposition in cotyledons of 14-day-old seedlings after a 24 h treatment with 500 nM flg22. Results are presented as means + SE. Asterisks indicate statistically significant difference between variants (one-way ANOVA, Tukey's HSD, followed by *post-hoc* pairwise comparison, $p<0.05$, n=18-36); ns, not significant. E, Resistance to *Pseudomonas syringae* pv. *tomato* DC3000 (*Pst*) *hrcC-*. 14-day-old seedlings were flooded with a *Pst hrcC-* suspension (OD_{600}=0.01) for 2 min, then the suspension was removed and after 2 days, the internal bacterial population was counted, log10 CFU/mg. Results are displayed as boxplots (center line, median; box limits, upper and lower quartiles; whiskers, 1.5x interquartile range; circles, individual values of independent samples). Different letters indicate statistically significant different values (one-way ANOVA, Tukey's HSD, $p<0.05$, n=4-14). DGK, diacylglycerol kinase.

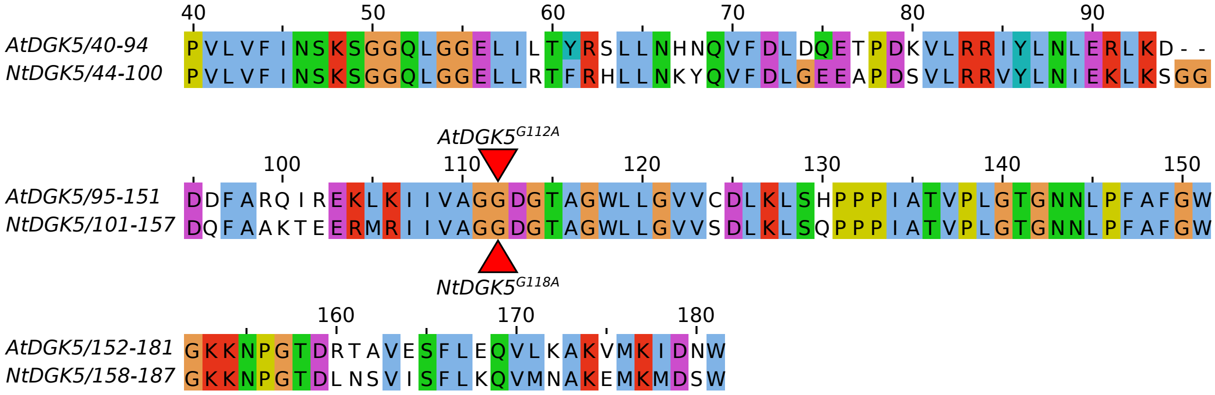

Supplemental Figure S8. Alignment of the DGK catalytic domain of DGK5 from Arabidopsis and tobacco. Arrowheads denote amino acid residues mutated in this study and in Scholz et al. (2022). DGK, diacylglycerol kinase.

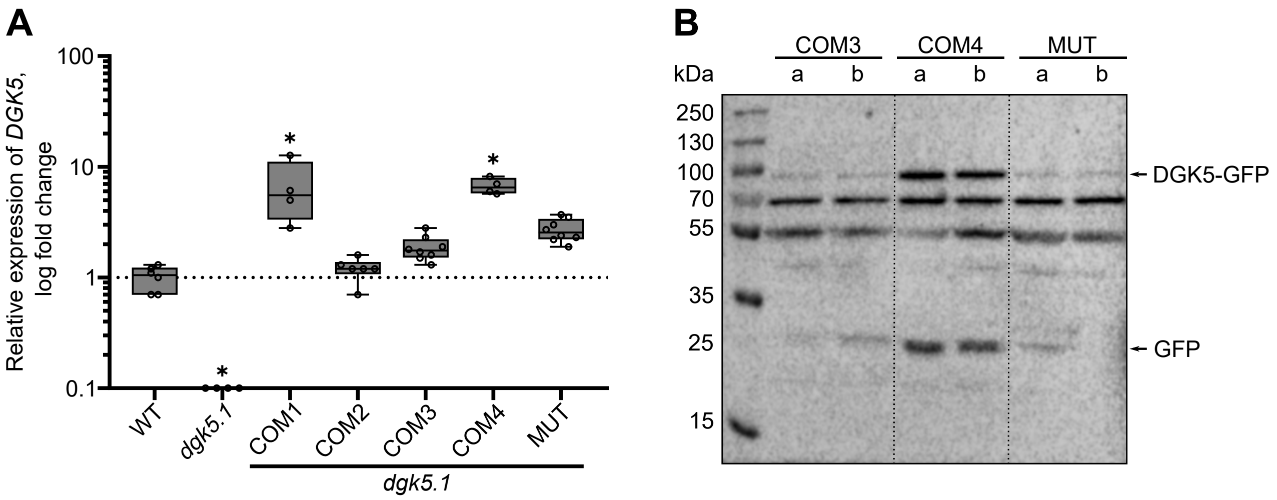

Supplemental Figure S9. Transcription of the *DGK5* gene and presence of fused proteins in tested independent complementation lines of *A. thaliana*. A, Total RNA was extracted from 7-day-old plants. *DGK5* transcription was measured using PQ11 and PQ12 primers and normalized to WT plant transcription levels. Results are displayed as boxplots (center line, median; box limits, upper and lower quartiles; whiskers, 1.5x interquartile range; circles, individual values of independent samples). Asterisks indicate significantly different values when compared to the WT, one-way ANOVA, $p<0.05$, n=4-6. B, Protein extracts were isolated from 7-day-old plants and samples were loaded according to measured protein concentrations. Western blots were probed with an anti-GFP antibody. Molecular weight of DGK5 fused to GFP and free GFP is 87 kDa and 27 kDa, respectively. Letters a, b stand for sample replicas. DGK, diacylglycerol kinase.

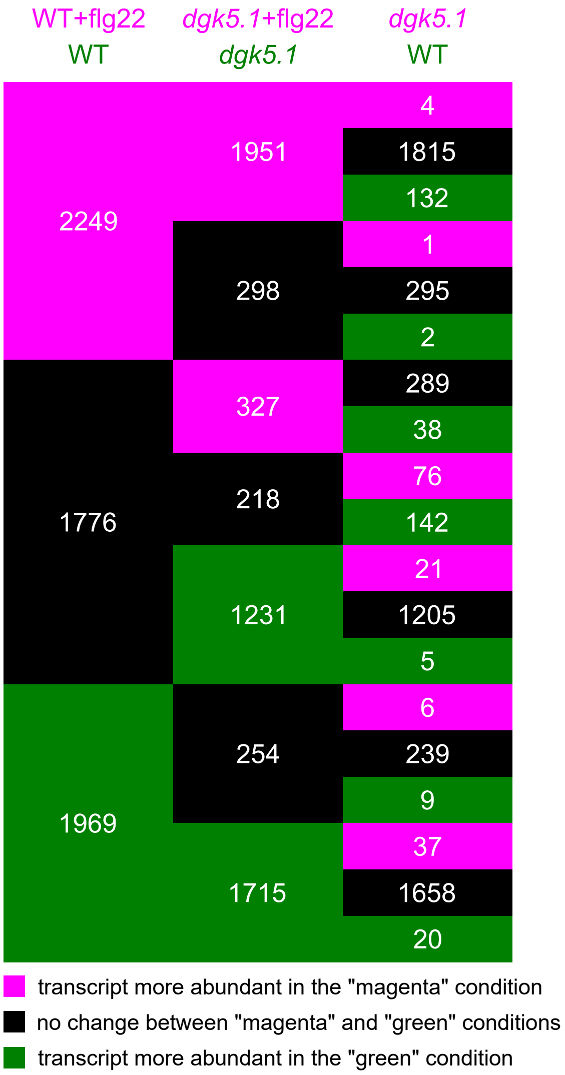

Supplemental Figure S10. Gene clustering according to transcriptomic data. Eleven-day-old seedlings (*dgk5.1* mutant or WT) were treated or not with 1 µM flg22 for 60 min and subjected to transcriptome analysis. Three comparisons were performed: “WT vs. WT+flg22”; “*dgk5.1* vs. *dgk5.1*+flg22”; “WT vs. *dgk5.1*”. Genes were clustered in 27 categories depending on their expression in the 3 comparisons. Some categories were not represented because no genes belonged to them. Magenta cells correspond to induced genes in the condition written in magenta, whereas green cells correspond to repressed genes in the condition written in magenta; black cells show no significant expression differences between the 2 conditions.

A

ANNOTATION	gene	*dgk5.1* vs WT	WT+flg22 vs WT	*dgk5.1*+flg22 vs *dgk5.1*
PR1 (*PATHOGENESIS-RELATED GENE 1*)	AT2G14610			
WRKY29 (*WRKY DNA-binding protein 29*); transcription factor	AT4G23550		2.96	3.18
WRKY30 (*WRKY DNA-binding protein 30*); transcription factor	AT5G24110	0.56	5.44	5.25
FRK1 (*FLG22-INDUCED RECEPTOR-LIKE KINASE 1*); kinase	AT2G19190		1.72	1.83

B

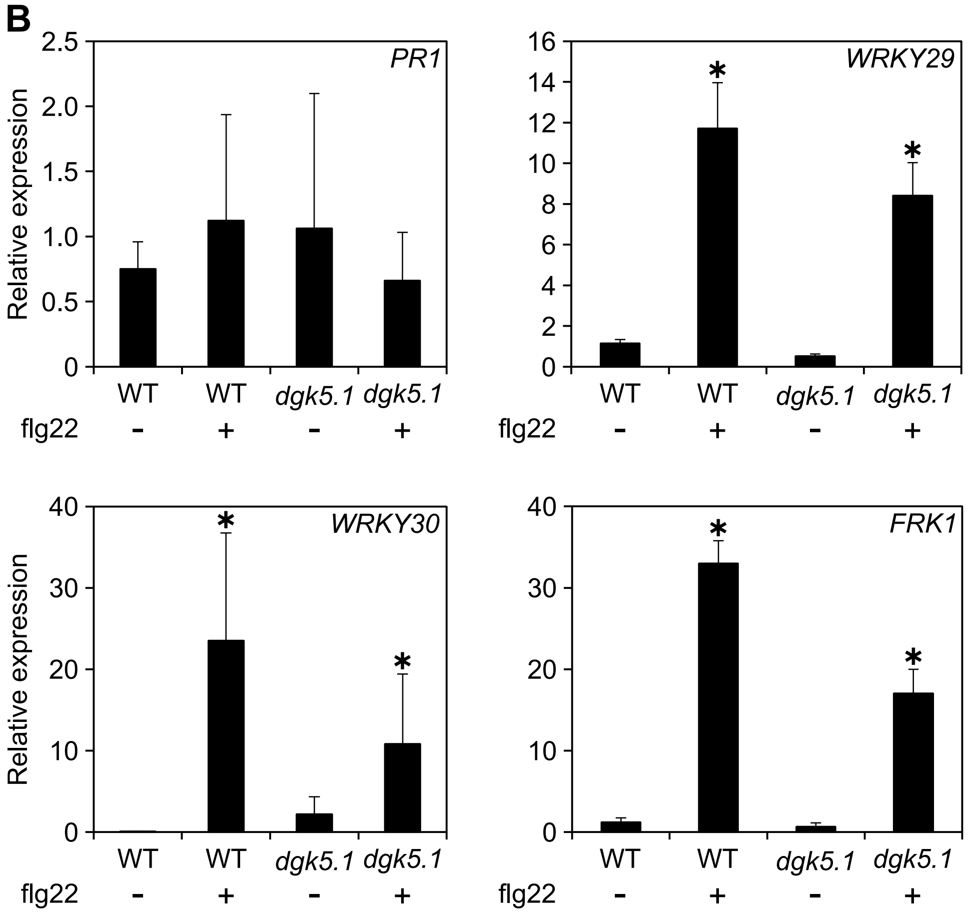

Supplemental Figure S11. Transcriptome validation. Results from CATMA microarray chips (A) were validated by RT-qPCR (B). Same RNA samples were used. *TIP41* (At4g34270) was used as a reference gene. Data are presented as means ± SE. Asterisks indicate samples that are different from controls, $p<0.05$, $n=3-4$, unpaired *t*-test.

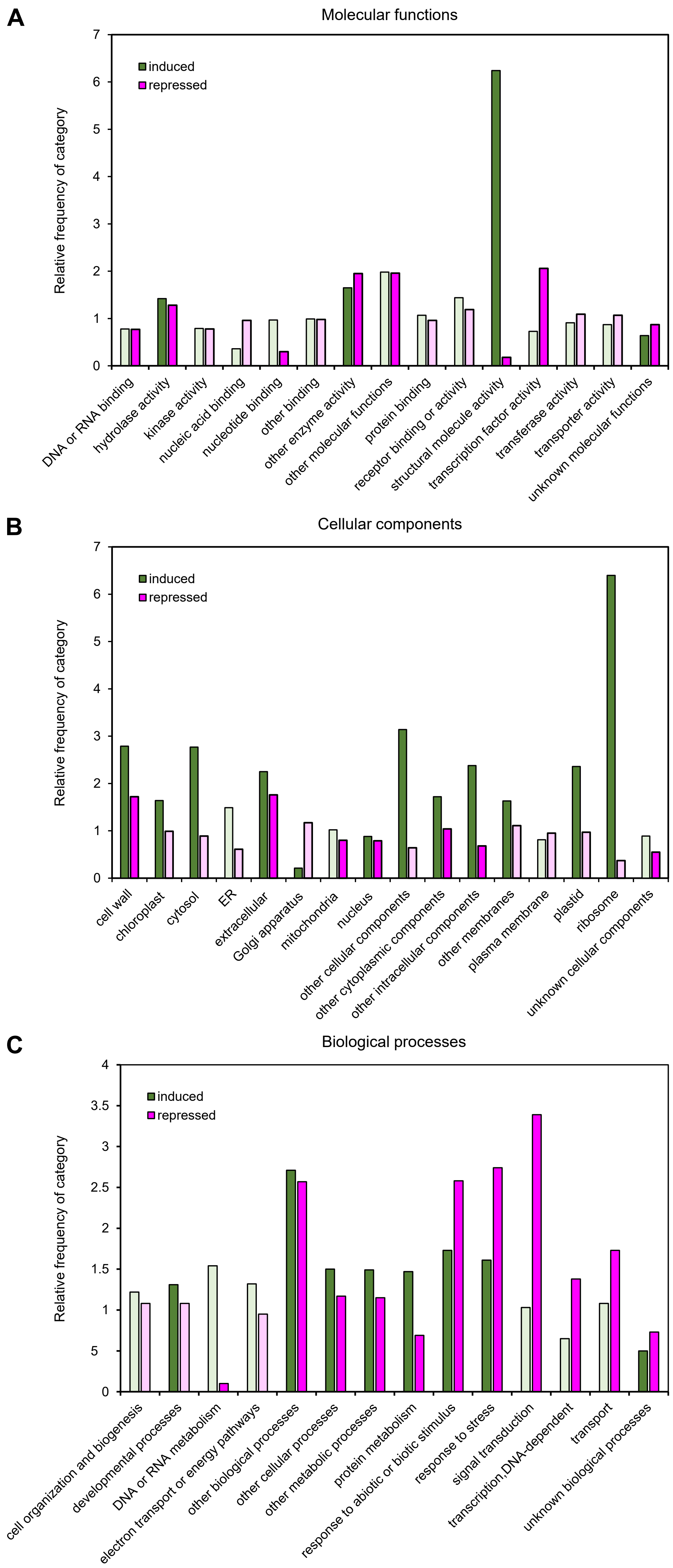

Supplemental Figure S12. Enrichment in GO categories in gene sets induced or repressed in *dgk5.1* versus WT. A, Molecular functions. B, Cellular components. C, Biological processes. Genes were classified using the Classification SuperViewer Tool developed by (Provart and Zhu, 2003). The classification source was set to Gene Ontology categories as defined by (Ashburner et al., 2000). The frequency of a category was normalized to that in the whole Arabidopsis set. The mean for 100 bootstraps of our input set was calculated to provide some idea about over- or under-representation reliability. Bars in bright color: statistically significant enrichment compared to whole Arabidopsis set.

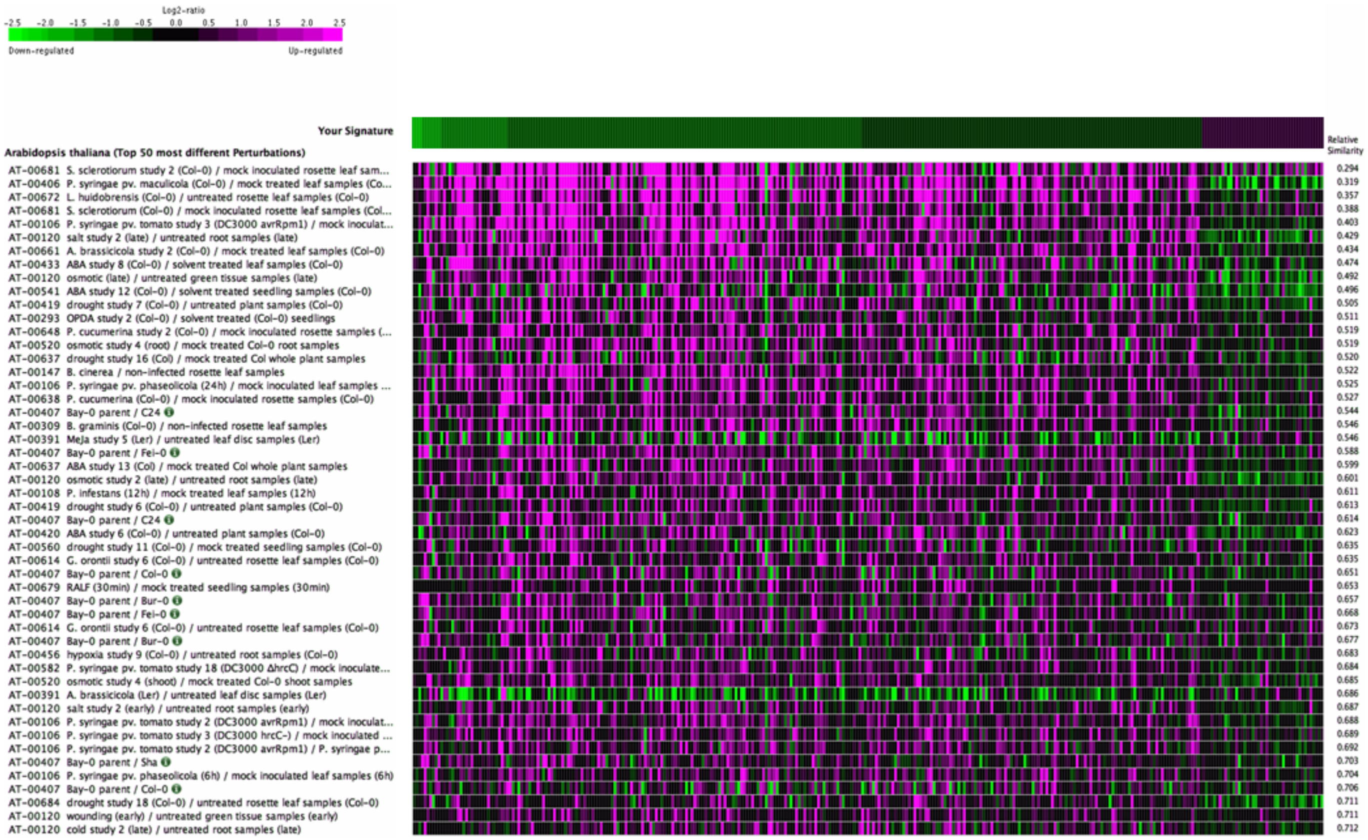

Supplemental Figure S13. Dissimilarity between the *dgk5.1*-responsive transcriptome and public transcriptome data. The set of genes altered (down- or up-regulated) in *dgk5.1* versus WT plants and their relative expression level as log2 ratio were used as a signature to search for experiments with the most different transcriptome changes. A similarity score, derived from Euclidean distance, was calculated by Genevestigator (Hruz et al., 2008) between our signature and each experiment of a set. Then a relative similarity score was calculated where a relative similarity score of 1 stands for a similarity between the input signature and an experiment that is the same as the average overall experiments of the set. The set of experiments we used were 423 curated experiments corresponding to the keywords “biotic, “hormone”, “stress” and “temperature” within the experiments classified as “Perturbations” in Genvestigator. The top 50 most different experiments compared to our signature are shown.

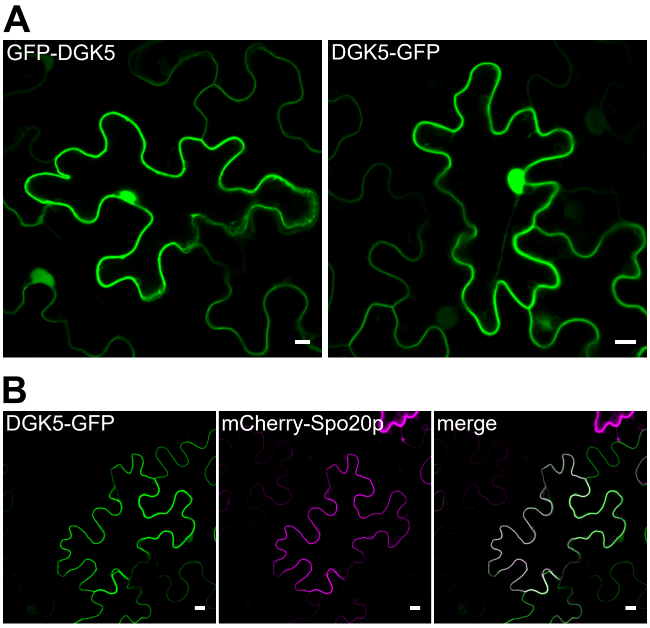

Supplemental Figure S14. DGK5 localization in *N. benthamiana*. A, GFP-DGK5 (left panel) and DGK5-GFP (right panel) transiently expressed in *N. benthamiana* leaf epidermis under the control of the 35S promoter. B, Co-localization of DGK5-GFP with the PA biosensor mCherry-NES-2xSpo20p-PABD. Samples were observed 24-48 h after transformation using a Zeiss LSM 880 confocal microscope and the 63X objective. DGK, diacylglycerol kinase. NES, nuclear exporting signal; PA, phosphatidic acid; PABD, PA-binding domain. Bars = 10 µm.

Supplemental Table S1. List of primers used in this study.

Label	Name	Sequence (5'- 3')
**RT-qPCR**		
PQ1	AtTIP41_FP	GTGAAAACTGTTGGAGAGAAGCAA
PQ2	AtTIP41_RP	TCAACTGGATACCCTTTCGCA
PQ3	AtPR1_FP	AGTTGTTTGGAGAAAGTCAG
PQ4	AtPR1_RP	GTTCACATAATTCCCACGA
PQ5	WRKY29_FP	CCATACCCAAGGAGTTATTAC
PQ6	WRKY29_RP	AACCGGCTAATGAGTTTC
PQ7	WRKY30_FP	GCAGCTTGAGAGCAAGAATG
PQ8	WRKY30_RP	AGCCAAATTTCCAAGAGGAT
PQ9	FRK1_FP	GCCAACGGAGACATTAGAG
PQ10	FRK1_RP	CCTTCAAGCTTTAATTGCGTC
PQ11	DGK5_fw2_FP	CCAGTGGCAGGACCTCCAC
PQ12	DGK5_fw2_RP	GGAATCTTGAAGGTATCCGCAG
**Genotyping of *dgk5.1* mutant**		
PG1	AtDGK5.1 LP_2018	TTCAGAGCACATGTGACCAAC
PG2	AtDGK5.1 RP_2018	TCCAATTCGGACATTTGTTTC
PG3	LB3_2018	TAGCATCTGAATTTCATAACCAATCTCGATACAC
**Cloning**		
PC1	pAtDGK5-F_SalI	ATAGTCGACCCGGTCTCCTTCACAATGAA
PC2	AtDGK5-3UTR-R_NotI	ATAGCGGCCGCGTATATGTTTGCTTTTTTTCCTTTCAATTAC
PC3	AtDGK5-NS-R_NotI	ATAGCGGCCGCAAGAGCACATGTGACCAACC
PC4	AtDGK5-G112A-F_forMP	CCATTATTATTAGGTTGCAGGAGCTGATGGCACTGCTGGG
PC5	AtDGK5-R_NdeI	AAACATATGCAGGCAAGTG
PC6	pAtDGK5-F_BstBI	CTTCTTCGAATTGTACGTTTATTG
PC7	AtDGK5-F_KpnI	ATAGGTACCATGGAGAAATACAACAGTTTATCAG
PC8	AtDGK5-S-R_NotI	ATAGCGGCCGCTCAGAGCACATGTGACCA
PC9	AtPLC2-F_SalI	ATAGTCGACAATGTCGAAGCAAACGTAC
PC10	AtPLC2-S-R_NotI	ATAGCGGCCGCTCACACAAACTCCACCT
PC11	AtRBOHD-F_KpnI	ATAGGTACCATGAAAATGAGACGAGGCAA
PC12	AtRBOHD-S-R_NotI	ATAGCGGCCGCCTAGAAGTTCTCTTTGTGGAAG
PC13	NES-GA5-F_Xba	ATATCTAGAAACTCAAATGAACTTGCCCTAAAACTTGCAGGGCTCGATAT<br>TAACAAGACCGAGGGTGCTGGTGCTGGTGCTGGTGCTGGTGCCGGCATG
PC14	Spo20-R_SpeI-int	ACCACTAGTCTTAGTGGCGTCATCGAAC
PC15	AtDGK5-F_ohHA	GATGTTCCAGATTACGCTATGGAGAAATACAACAGTTTATCAG
PC16	pTNT-HA-F_SalI	AAGTCGACGCCGCCACCATGTACCCATACGATGTTCCAGATTACGCTATG
